# Limited Foxp3^+^ Regulatory T Cells Response During Acute *Trypanosoma cruzi* Infection Is Required to Allow the Emergence of Robust Parasite-Specific CD8^+^ T Cell Immunity

**DOI:** 10.3389/fimmu.2018.02555

**Published:** 2018-11-05

**Authors:** Cintia L. Araujo Furlan, Jimena Tosello Boari, Constanza Rodriguez, Fernando P. Canale, Facundo Fiocca Vernengo, Santiago Boccardo, Cristian G. Beccaria, Véronique Adoue, Olivier Joffre, Adriana Gruppi, Carolina L. Montes, Eva V. Acosta Rodriguez

**Affiliations:** ^1^Departamento de Bioquímica Clínica, Facultad de Ciencias Químicas, Universidad Nacional de Córdoba, Córdoba, Argentina; ^2^Centro de Investigaciones en Bioquímica Clínica e Inmunología, CONICET, Córdoba, Argentina; ^3^Institut National de la Santé et de la Recherche Médicale, Toulouse, France; ^4^Centre National de la Recherche Scientifique, Toulouse, France; ^5^Centre de Physiopathologie de Toulouse Purpan, Université de Toulouse, Université Paul Sabatier, Toulouse, France

**Keywords:** regulatory T (Treg) cells, *Trypanosoma cruzi*, immunity, CD8 cytotoxic T cells^+^, pathogenesis

## Abstract

While it is now acknowledged that CD4^+^ T cells expressing CD25 and Foxp3 (Treg cells) regulate immune responses and, consequently, influence the pathogenesis of infectious diseases, the regulatory response mediated by Treg cells upon infection by *Trypanosoma cruzi* was still poorly characterized. In order to understand the role of Treg cells during infection by this protozoan parasite, we determined in time and space the magnitude of the regulatory response and the phenotypic, functional and transcriptional features of the Treg cell population in infected mice. Contrary to the accumulation of Treg cells reported in most chronic infections in mice and humans, experimental *T. cruzi* infection was characterized by sustained numbers but decreased relative frequency of Treg cells. The reduction in Treg cell frequency resulted from a massive accumulation of effector immune cells, and inversely correlated with the magnitude of the effector immune response as well as with emergence of acute immunopathology. In order to understand the causes underlying the marked reduction in Treg cell frequency, we evaluated the dynamics of the Treg cell population and found a low proliferation rate and limited accrual of peripheral Treg cells during infection. We also observed that Treg cells became activated and acquired a phenotypic and transcriptional profile consistent with suppression of type 1 inflammatory responses. To assess the biological relevance of the relative reduction in Treg cells frequency observed during *T. cruzi* infection, we transferred *in vitro* differentiated Treg cells at early moments, when the deregulation of the ratio between regulatory and conventional T cells becomes significant. Intravenous injection of Treg cells dampened parasite-specific CD8^+^ T cell immunity and affected parasite control in blood and tissues. Altogether, our results show that limited Treg cell response during the acute phase of *T. cruzi* infection enables the emergence of protective anti-parasite CD8^+^ T cell immunity and critically influences host resistance.

## Introduction

Regulatory T (Treg) cells, defined by the expression of the lineage transcription factor forkhead box P3 (Foxp3), are able to suppress most immune cells (1) and their suppressive function is crucial for immune homeostasis and prevention of autoimmunity (2). In addition, Treg cells were shown to be critical mediators in the modulation of host-microbe interaction during infections and to play beneficial or deleterious roles in host resistance (3). Several chronic bacterial infections, such as tuberculosis (4) and leprosy (5) promote the accumulation and preferential migration of Treg cells to target tissues, where these cells exert regulatory effects that compromise protective responses and favor bacterial persistence (6). During viral infections, the role of this cell subset appears to be different between acute and chronic infections and even to change during the same infection with the transition from acute to chronic stages. In this way, Treg cells have been shown to coordinate early protective immunity to mucosal Herpes Simplex Virus (7, 8) and pulmonary Respiratory Syncytial Virus (9, 10), and to sustain memory CD8^+^ T cell immunity to West Nile virus (11). In contrast, there is by far more evidence that Treg cells accumulate during certain viral infections and dampen adaptive immune responses, specially CD8^+^ T cell immunity, promoting virus establishment and infection chronicity (12, 13). Thus, Treg cells seem to exert a general beneficial role by limiting exuberant immune responses and the consequent excessive inflammation and immunopathology even at the expense of reducing viral control (12, 14, 15). Similar scenarios have been reported in the course of parasitic infections (16). During leishmaniasis (17) and malaria (18), Treg cells have been shown to limit the magnitude of effector responses, resulting in failure to adequately control infection (19–21). In contrast, these cells favor host resistance during infections with *S. mansoni* and *T. gondii* by restraining collateral tissue damage caused by vigorous anti-parasite immune responses (22–24). In addition, relative or absolute reduction in Treg cell numbers during acute infections with *T. gondii* (23, 25), *L. monocitogenes* (25), vaccinia virus (25) and LCMV clone Armstrong (26) supports the emergence of CD4^+^ and CD8^+^ T cell immunity. Therefore, the impact of Treg cells in the outcome of an infection is expected to be different depending on the pathogen, timing and affected tissues, while their manipulation may open up new avenues for therapeutic strategies.

Chagas disease (American Trypanosomiasis) is a life-threatening illness caused by the protozoan parasite *T. cruzi* (27). Last estimates calculated an infected population of about 6 million in endemic areas of Latin America and several hundred thousand worldwide, with more than 70 million people living at risk of infection and 40,000 new cases diagnosed per year (28). Host resistance to *T. cruzi* depends on both innate and adaptive immune responses which are triggered early during infection (29–31). Macrophages, dendritic cells, natural killer cells and B and T lymphocytes act in concert to control parasite replication but are not able to completely eradicate the pathogen (32). In particular, parasite-specific antibodies and CD8^+^ T cells together with a type I response with production of IFN-γ and TNF are critical for host resistance (32). Nevertheless, exuberant production of these inflammatory cytokines has been associated with tissue damage, immunopathology and disease severity in mice and humans (33–36), supporting the notion that regulatory responses greatly impact in the final outcome of *T. cruzi* infection. In this context, many studies aimed to understand the role of Treg cells during the progression of this parasitic infection, reporting often contradictory results. The frequency and functionality of Treg cells were shown to be increased in the peripheral blood of infected patients that presented less severe chronic disease (37–40), suggesting a beneficial role for this cell subset during human Chagas disease. On the other hand, experimental models reported protective (41, 42), limited (43, 44) and also deleterious (45) effects for Treg cells during *T. cruzi* infection. However, none of these studies addressed the kinetics or the phenotypical and functional features of the regulatory response, and more importantly, all of them targeted Treg cells by non-specific approaches. These technical limitations have delayed an accurate characterization of Treg cell responses during *T. cruzi* infection and, therefore, prevented any rational manipulation of this subset in order to modulate the outcome of the chronic disease.

In this manuscript, we took advantage of Foxp3-EGFP reporter mice to comprehensively determine the magnitude and quality of the Treg cell response triggered by *T. cruzi* infection. In addition, adoptive transfer experiments of *in vitro* differentiated Treg cells allowed us to establish the biological role of this subset in the regulation of protective immunity and parasite control.

## Materials and methods

### Mice

Mice used for experiments were sex- and age-matched. C57BL/6 and BALB/c wild type mice were obtained from School of Veterinary, La Plata National University (La Plata, Argentina). CD45.1 C57BL/6 mice (B6.SJL-Ptprc^a^ Pepc^b^/BoyJ), Foxp3-EGFP reporter mice (B6.Cg-Foxp3^tm2Tch^/J), IL-6 deficient mice (B6.129S2-Il6^tm1Kopf^/J) and Caspase-1/11 deficient mice (B6N.129S2-Casp1^tm1Flv^/J) were obtained from The Jackson Laboratories (USA). IFNAR deficient mice (Ifnar1^tm1Ag^) were obtained from Pasteur Institute (Paris, France) (46). Animals were bred in the animal facility of the Facultad de Ciencias Químicas, Universidad Nacional de Córdoba, and housed under a 12 h: 12 h light-dark cycle with food and water *ad libitum*.

### Parasites and experimental infection

Bloodstream trypomastigotes of the Tulahuén strain of *T. cruzi* were maintained in BALB/c mice by serial passages each 10–11 days. For experimental infection, 7–10 weeks-old mice were inoculated intraperitoneally with 0.2 ml PBS containing 5 × 10^3^ trypomastigotes. Alternatively, doses of 500 and 5 × 10^4^ trypomastigotes were used as indicated.

Live *T. cruzi* trypomastigotes (Tulahuén) were obtained from the extracellular medium of infected monolayers of Vero cells, as previously described (47).

### Quantification of parasite numbers in blood and parasite burden in tissues

Parasitemia was monitored by counting the number of viable trypomastigotes in blood after lysis with a 0.87% ammonium chloride buffer. Abundance of *T. cruzi* satellite DNA in tissues was used to determine parasite burden. Genomic DNA was purified from 50 μg of tissue (spleen and liver) using TRIzol Reagent (Life Technologies) following manufacturer's instructions. Satellite DNA from *T. cruzi* (GenBank AY520036) was quantified by real time PCR using specific Custom Taqman Gene Expression Assay (Applied Biosystem). Primers and probes sequences were previously described by Piron et al (48). A sample was considered positive for *T. cruzi* target when C_T_ < 45. Abundance of satellite DNA from *T. cruzi* was normalized to GAPDH abundance (Taqman Rodent GAPDH Control Reagent, Applied Biosystem), quantified through the comparative C_T_ method and expressed as arbitrary units.

### Cell preparation, purification, and culture

Blood was collected using heparin as anticoagulant. Spleens, thymi, livers and mesenteric and inguinal lymph nodes were obtained and pressed through a tissue strainer to obtain cell suspensions. Bone marrow cells were isolated by flushing femurs and tibias with PBS 2% FBS. Liver infiltrating cells were obtained after 25 min centrifugation (600 g) in a 35 and 70% bilayer of Percoll (GE Healthcare) gradient. Erythrocytes in bone marrow, spleen, thymus and liver cell suspensions were lysed for 3 min in ammonium chloride-potassium phosphate buffer. Cell numbers were counted in Turk's solution using a Neubauer chamber.

CD4^+^ and CD8^+^ T cells were isolated from pooled splenic suspensions by magnetic negative selection using EasySep™ Mouse CD4^+^ or CD8^+^ T Cell Isolation Kits, respectively (StemCell Technologies). After surface staining, Treg, Tconv and naïve CD4^+^ T cells were further purified from CD4^+^ T cell suspensions by cell sorting with a FACSAria II (BD Biosciences) using the following gating strategy: Treg cells: CD4^+^ Foxp3-GFP^+^ CD25int/hi; Tconv cells: CD4^+^ Foxp3-GFP^−^ CD25^−/+^; and naïve CD4^+^ T cells: CD4^+^ Foxp3-GFP^−^ CD25^−^ CD62Lhi CD44lo. Cells were cultured in RPMI 1640 medium (Gibco, Invitrogen) supplemented with 2 mM glutamine (Gibco, Invitrogen), 55 μM 2-ME (Gibco, Invitrogen), and 40 μg/ml gentamicin (Veinfar Laboratories) containing 10% heat inactivated FBS (Gibco or Natocor).

For *in vitro* Treg cell differentiation, naïve CD4^+^ T cells or total splenocytes were stimulated at a cell density of 2 × 10^5^/well during 3–4 days in 96-well cell culture plates coated with 2 μg/mL anti-CD3 (eBioscience) and 1 μg/mL anti-CD28 (BD Biosciences), in presence or absence of a Treg cell differentiation cocktail containing 100 U/mL mrIL-2 (RandD), 5 ng/mL rTGF-β1 (eBioscence) and 13.3 nM all trans-Retinoic Acid (Sigma). In the experiments aimed to evaluate the inhibition of Treg cell induction, live *T. cruzi* parasites were added in different ratios as described in the corresponding legend for figure.

### Biochemical determinations and quantification of cytokines in plasma

Blood was centrifuged for 8 min at 3,000 rpm and plasma was collected. Plasma samples were sent to Biocon Laboratory (Córdoba, Argentina) for quantification of GOT, GPT, LDH and CPK by UV kinetic methods, and glucose by kinetic/colorimetric method in a Dimension RXL Siemens analyzer. Plasma levels of different cytokines were determined in our laboratory using LEGENDplex™ Multi-Analyte Flow Assay Kit (Biolegend) for Mouse Th Cytokine and Inflammation Panels according to manufacturer's instructions.

### Antibodies and flow cytometry

For surface staining, cell suspensions were incubated with fluorochrome labeled-antibodies in PBS 2% FBS for 20 min at 4°C. Flow cytometry and/or cell sorting were performed with a combination of the following antibodies: anti-CD4 APC, APC-eFluor-780 or PerCP-eFluor 710 (all clone GK1.5), anti-CD25 PE-Cy7 (P61.5), anti-CD3 PerCP-Cy5.5 (145-2C11), anti-CD8a FITC, PE, PE-Cy7 or PerCP-Cy5.5 (53-6.7), anti-CCR7 PerCP-Cy5.5 (4B2), anti-CD103 PE (2-E7), anti-CD127 PerCP-eFluor-710 (eBioSB/199), anti-CD39 eFluor-660 (24DMS1), anti-CD44 APC-Cy7 (IM7), anti-CD45.2 PerCP-Cy5.5 (104), anti-CD62L PerCP-Cy5.5 (MEL-14), anti-CD73 biotin (eBioTY/11-8), anti-CTLA-4 PE (UC10.4B9), anti-CXCR3 PE (CXCR3-173), anti-FR4 PE (eBio12a5), anti-GARP APC (YGIC86), anti-OX40 APC (OX-86) and anti-PD-1 PE (RMP1-30) from eBioscience; anti-CD4 AlexaFluor700 (GK1.5), anti-CD45.1 APC-Cy7 (A20), anti-GITR PE-Cy7 (DTA-1), anti-LAG-3 PerCP-Cy5.5 (C9B7W), anti-KLRG1 PE (2F1/KLRG) and anti-LAP PE (TW7-20B9) from Biolegend; and anti-CD127 biotin (B12-1) from BD Biosciences. To detect biotinilated antibodies, Streptavidin APC (Biolegend) or PerCP-Cy5.5 (eBioscience) were used. To identify *T. cruzi* specific CD8^+^ T cells, cell suspensions were incubated with an H-2K^b^ *T. cruzi* trans-sialidase amino acids 569-576 ANYKFTLV (TSKB20) APC-labeled Tetramer (NIH Tetramer Core Facility) for 20 min at 4°C before further surface staining with additional antibodies. Blood was directly incubated with the indicated antibodies and erythrocytes were lysed with a 0.87% NH_4_Cl buffer previously to acquisition. To assess apoptosis, cells were stained for Annexin V PE (Biolegend) and 7-AAD (BD Biosciences) according to the manufacturer's specifications.

For detection of transcription factors, cells were first stained on surface, washed and then fixed, permeabilized and stained with Foxp3/Transcription Factor Staining Buffers (eBioscience) according to eBioscience One-step protocol for intracellular (nuclear) proteins. The following antibodies were used for intracellular staining: anti-Foxp3 PE, PerCP-Cy5.5 or APC (FJK-16s), anti-T-bet PerCP-Cy5.5 or PE-Cy7 (eBio4B10), and anti-Ki-67 APC (SolA15) from eBioscience.

For intracellular cytokine staining, 2 × 10^6^ cells per well were cultured in 200 μL supplemented RPMI 1640 medium and stimulated during 2–5 h at 37°C with 50 ng/mL PMA and 1 μg/mL ionomycin (Sigma-Aldrich) in the presence of Brefeldin A and/or Monensin (eBioscience). After surface staining, cells were fixed and permeabilized with Intracellular Fixation & Permeabilization Buffer Set (eBioscience) or BD Cytofix/Cytoperm and Perm/Wash (BD Biosciences) following manufacturers' indications. Cells were then labeled with anti-IL-10 APC (JES5-16E3), anti-IFN-γ APC or PE (XMG1.2) and anti-IL-17A PE (eBio17B7) from eBioscience; anti-IL-10 PE (JES5-16B3) and anti-TNF PerCP-Cy5.5 (MP6-XT22) from Biolegend.

All samples were acquired on a FACSCanto II (BD Biosciences) and data were analyzed with FlowJo software.

### Adoptive cell transfer

For *in vivo* pTreg cells induction experiments, Tconv cells were purified from non-infected Foxp3-EGFP CD45.2 donors as described above. Two millions cells were then intravenously injected in the retro-orbital sinus of CD45.1 recipient mice, which were simultaneously infected with the usual dose of *T. cruzi*. Non-infected and/or non-transferred mice were used as controls. Conversion of injected cells (Foxp3-GFP^−^) into Treg cells (Foxp3-GFP^+^) was assessed by flow cytometry in different organs 19 days after treatment.

To assess the biological relevance of reduced Treg cells frequency, 1 × 10^6^ *in vitro* differentiated Treg cells generated from Foxp3-EGFP mice were intravenously transferred into CD45.1 recipient mice at 11 days post-infection (dpi). The effect of Treg cell adoptive transfer was evaluated 7 days later.

### RNAseq

Treg cells were purified as described above from the spleens of non-infected and 22-days infected Foxp3-EGFP mice and immediately lysed with QIAzol reagent (Qiagen). Total RNA was extracted by using the RNeasy Micro Kit (Qiagen) and its quality was assessed on a 2100 Bioanalyzer (Agilent Technologies). RNA-seq libraries were then prepared according to the TruSeq Stranded Total RNA Sample protocol (Illumina). Quality controls of the libraries were performed by standard methods, including quantification by Qubit (Thermo Fisher Scientific) and assessment of size distribution using a 2100 Bioanalyzer. Samples were indexed and sequenced on an Illumina HiSeq 3000 (paired-end reads of 150 bp). Bioinformatic analysis of sequenced Reads was performed as described elsewhere (49). *P*-values <0.1 (adjusted for multiple testing by Benjamini–Hochberg procedure) were considered as the cutoff for significantly differentially expressed genes. Gene Set Enrichment Analyses (GSEA) for Th1, Th2, and Th17 signature were performed using specific gene sets selected from several publications as indicated in Supplementary Table 1 and depicted in heat maps of **Figure 4E**. NCBI Sequence Read Archive accession code: SRP145339.

### Statistics

Statistical significance of mean values comparisons was analyzed by *t*-test, Mann Whitney test, One-way ANOVA or Kruskal-Wallis test as indicated. According to the absolute values of r, the Spearman correlation was interpreted as strong (*r* = 0.7–0.9), good (*r* = 0.50–0.70), moderate (*r* = 0.30–0.50), and poor (*r* < 0.30) (50–52). Statistical analysis was performed using GraphPad Prism 7.0 software. *P*-values ≤0.05 were considered significant.

## Results

### Treg cell frequency is decreased during the progression of *T. cruzi* infection

**Figure 1:**
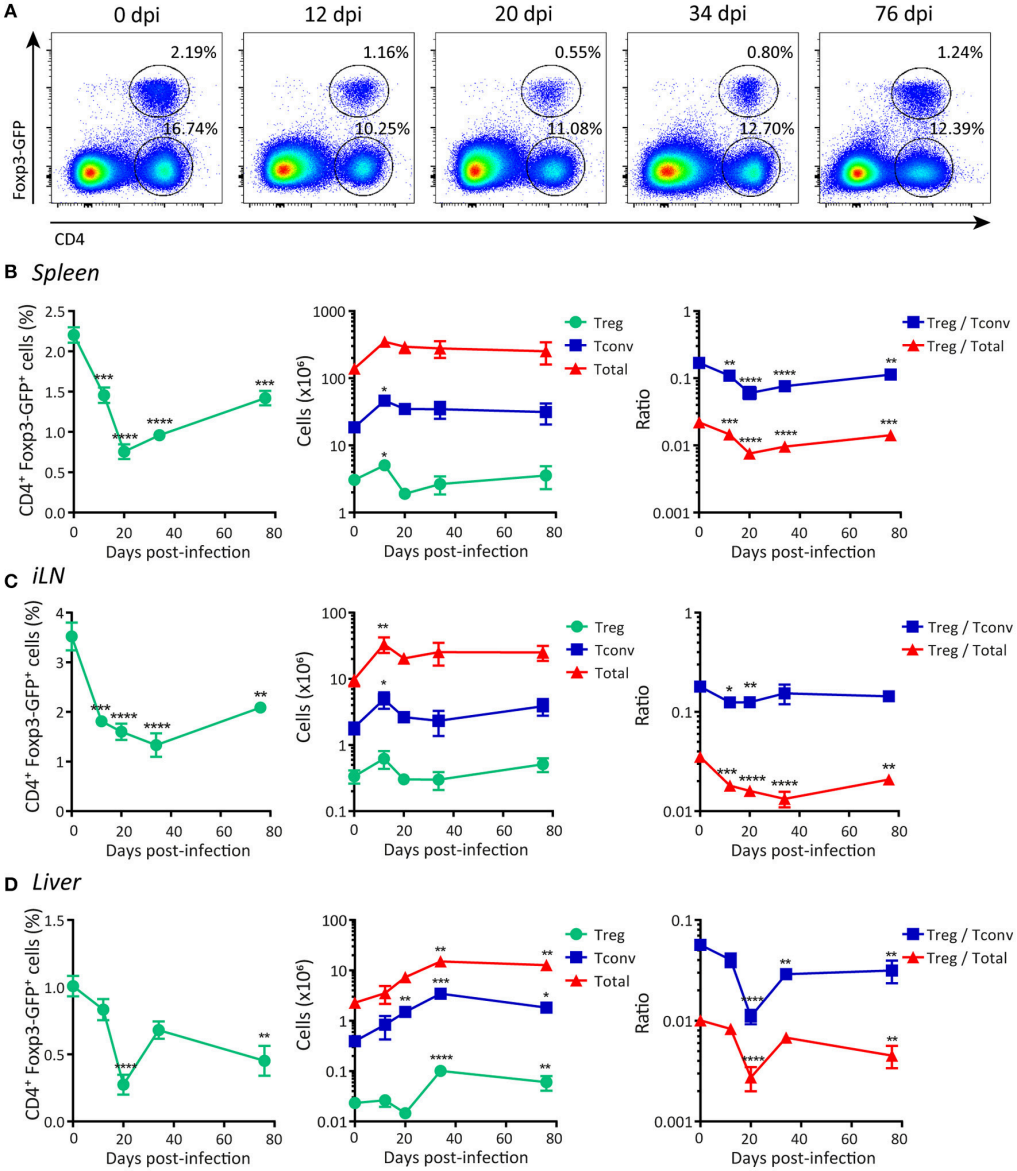
Frequency of Foxp3^+^ regulatory T cells is reduced in peripheral organs during acute *T. cruzi* infection. **(A)** Flow cytometry representative dot plots showing Foxp3-GFP expression and CD4 staining in the spleen of *T. cruzi* infected Foxp3-EGFP mice at different days post-infection (dpi). **(B–D)** Graphs showing CD4^+^ Foxp3-GFP^+^ Treg cell frequencies (left panel), absolute numbers of Treg cells, CD4^+^ Foxp3-GFP^−^ (Tconv) cells and total leukocytes (middle panel), and the ratios of Treg cells to Tconv and to total cells in the spleens **(B)**, inguinal lymph nodes **(C)**, and liver infiltrating leucocytes **(D)** of *T. cruzi* infected Foxp3-EGFP mice at different dpi. Data are presented as mean ± SEM, *n* = 3–11 animals per dpi. Data are representative of at least three independent experiments. *P*-values were calculated by One way ANOVA with Dunnett's multiple comparisons test or Kruskal-Wallis with Dunn's correction (Tconv and total cells absolute numbers in liver only). ^*^P ≤ 0.05, ^**^P ≤ 0.01, ^***^P ≤ 0.001 and ^****^P ≤ 0.0001.

As an initial step to characterize Treg cell responses, we evaluated how *T. cruzi* infection affected the frequency and absolute numbers of Treg cells and other immune cell subsets. Using Foxp3 reporter mice, we determined that acute *T. cruzi* infection resulted in a significant decrease in the percentage of Treg cells (CD4^+^ Foxp3-GFP^+^) in the spleen (Figure 1A). This decrease in the frequency of splenic Treg cells was statistically significant after 12 days post-infection (dpi) and remained low even at the end of the acute phase (76 dpi; Figure 1B, left panel). Of note, the absolute numbers of Treg cells remained unchanged or were transiently increased at low extent while the numbers of CD4^+^ Foxp3-GFP (Tconv) cells and total spleen leukocytes were augmented by several orders of magnitude (Figure 1B, middle panel). Consequently, the ratios between the numbers of Treg cells and Tconv cells or total cells were significantly decreased along the infection (Figure 1B, right panel). A similar picture was observed in secondary lymphoid organs, such as inguinal lymph nodes (iLN) (Figure 1C) as well as in the liver, a target tissue with intense parasite replication and immune cell infiltration during the acute phase of *T. cruzi* infection (53) (Figure 1D). This reduction in the frequency of Treg cells and/or the ratio of Treg cell numbers to Tconv and total cell numbers were also detected in blood and bone marrow (Supplementary Figures 1A,B). Altogether, these results indicate that, in contrast to most chronic infections (6), Treg cells do not significantly accumulate in secondary lymphoid organs or liver during *T. cruzi* infection.

Next, we evaluated Treg cells frequencies and numbers in organs associated with the development of this subset, e.g., thymus and gut-associated mesenteric lymph nodes (mLN) (54). We determined that the percentage of Treg cells as well as the ratio between the numbers of this population and other cell subsets in the thymus were significantly increased during *T. cruzi* infection (Supplementary Figure 1C, left and right panels), likely as consequence of conserved Treg cells numbers together with a marked reduction in the number of total thymocytes (Supplementary Figure 1C, middle panel). In mLN, the numbers of Treg cells showed an initial oscillation (increase followed by decrease) after infection while the frequency and the ratio of Treg cells to Tconv numbers exhibited an early increase, but all these parameters remained unaltered afterwards (Supplementary Figure 1D).

### The frequency of treg cells is negatively correlated with markers of infection progression and the development of effector immune responses

**Figure 2:**
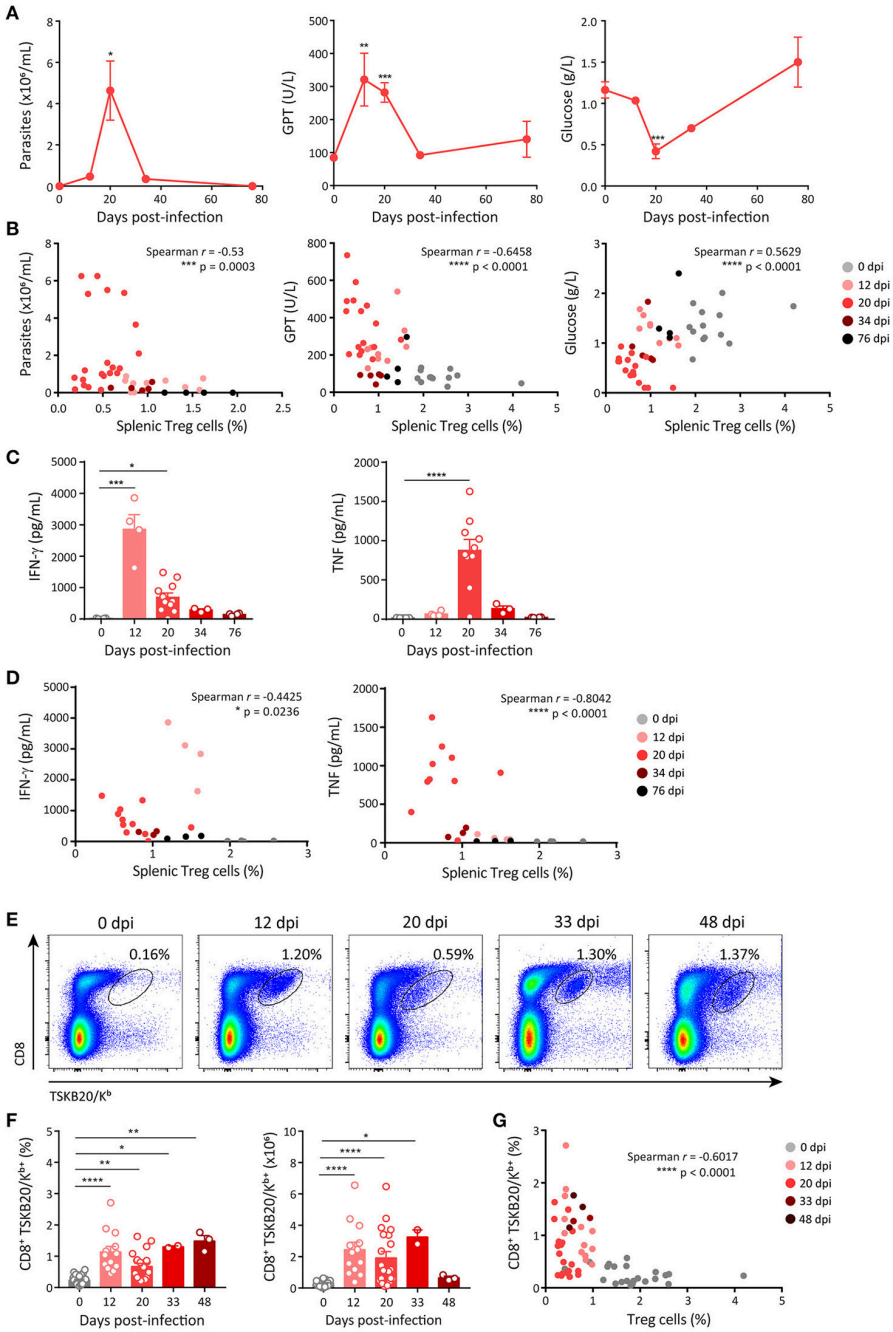
Frequency of Treg cells inversely correlates with markers of acute disease progression and the emergence of effector immune responses. **(A)** Parasitemia (left panel) in blood, activity of glutamate-pyruvate transaminase (GPT) (middle panel) and glucose concentration (right panel) in plasma of *T. cruzi* infected Foxp3-EGFP mice at different dpi. **(B)** Scatter plots showing the relation between splenic Treg cell frequencies and each of the parameters shown in **(A)**. **(C)** Concentration of effector cytokines in plasma of *T. cruzi* infected Foxp3-EGFP mice at different dpi. **(D)** Scatter plots showing the relation between Treg cell frequencies in the spleen and plasma level of effector cytokines. **(E)** Representative dot plots for CD8 and TSKB20/K^b^ staining in the spleen of *T. cruzi* infected Foxp3-EGFP mice at different dpi. **(F)** Percentage (left) and absolute numbers (right) of parasite-specific CD8^+^ T cells in the spleen *T. cruzi* infected Foxp3-EGFP mice at different dpi. **(G)** Scatter plots showing the relation between frequencies of Treg cells and parasite-specific CD8^+^ T cells in the spleen of mice shown in **(F)**. In **(A)** data are presented as mean ± SEM of *n* = 3–11 animals. In **(B–D,F,G)** each circle or dot represents one animal. In **(C,F)** the bars show the mean + SEM of each parameter. Spearman *r* correlation coefficient and significance of the correlation are indicated inside the corresponding graphs. Data were pooled from 1 to 6 experiments according to the dpi and the determination. *P*-values were calculated by One way ANOVA with Dunnett's multiple comparisons test in **(A)** and Kruskal-Wallis with Dunn's correction in **(C,F)**. ^*^P ≤ 0.05, ^**^P ≤ 0.01, ^***^P ≤ 0.001 and ^****^P ≤ 0.0001.

We next aimed at studying whether the changes in the frequency of Treg cells during *T. cruzi* infection showed any correlation with disease progression and/or the magnitude of effector immune responses. As markers of progression in the infection by *T. cruzi* we evaluated parasitemia and the levels of different biochemical parameters used to assess general health and tissue damage. As depicted in Figure 2A, parasite numbers in blood were low but detectable at 12 dpi, peaked around 20 dpi, diminished by 34 dpi and became undetectable later on. Similar kinetics was observed when measuring the activity of enzymes that reflect tissue damage (i.e., GPT, LDH, GOT, and CPK; Figure 2A, middle panel; Supplementary Figure 2A). In contrast, the concentration of plasma glucose, whose decrease is generally associated with acute infection (55), was reduced alongside the progression of infection, showing the lowest level around 20 dpi and reaching normal levels afterwards (Figure 2A, right panel). Remarkably, changes in the levels of all these parameters showed statistically significant strong, good or moderate correlations with the frequencies of Treg cells in the spleen (Figure 2B; Supplementary Figure 2B). These correlations were direct for glucose concentration and inverse for parasitemia and enzymatic activity in plasma.

To assess the effector immune response, we quantified the plasma concentration of effector cytokines known to be protective during *T. cruzi* infection, such as IFN-γ and TNF (32). The concentration of IFN-γ showed a marked increase by 12 dpi and diminished later on, while the kinetics of TNF production was slower, showing the peak of plasma concentration at 20 dpi (Figure 2C). In addition, we quantified other effector cytokines, such as IL-6, IL-1β, and IL-2. The concentration of IL-6 was significantly increased by 12 dpi, peaked at 20 dpi and decreased afterwards whereas the concentrations of IL-1β and IL-2 remained unchanged along the infection (Supplementary Figure 2C). The concentrations of IFN-γ, TNF and IL-6, but not those of IL-1β and IL-2, showed significant strong, good or moderate inverse correlations with the frequency of Treg cells in spleen (Figure 2D; Supplementary Figure 2D). We also evaluated the presence of parasite-specific CD8^+^ T cell immunity as a component of the cellular effector immune response known to be critical for parasite control (31). CD8^+^ T cells specific for the immunodominant *T. cruzi* peptide TSKB20 were identified in the spleen of *T. cruzi* infected mice using tetramers (Figure 2E). We found that the frequency and absolute numbers of TSKB20-specific CD8^+^ T cells increased between 12 and 48 dpi (Figures 2E,**F**). Similar to effector cytokines, the frequency of TSKB20-specific CD8^+^ T cells showed a good inverse correlation with the frequency of Treg cells (Figure 2G).

### Limited expansion and reduced accrual of peripheral treg cells during acute *T. cruzi* infection

In steady state conditions, the size of the Treg cell population is maintained by a dynamic process, through a balance among development, proliferation and apoptosis (56). During infections, these events may be altered leading to the accumulation, constriction or dysfunction of Treg cells and consequently, modulating the effector immune response and the host-microbe interaction (57). In this context, we aimed to evaluate whether the reduction of Treg cell frequency during *T. cruzi* infection was a consequence of alterations in the mechanisms that sustain Treg cell homeostasis.

**Figure 3:**
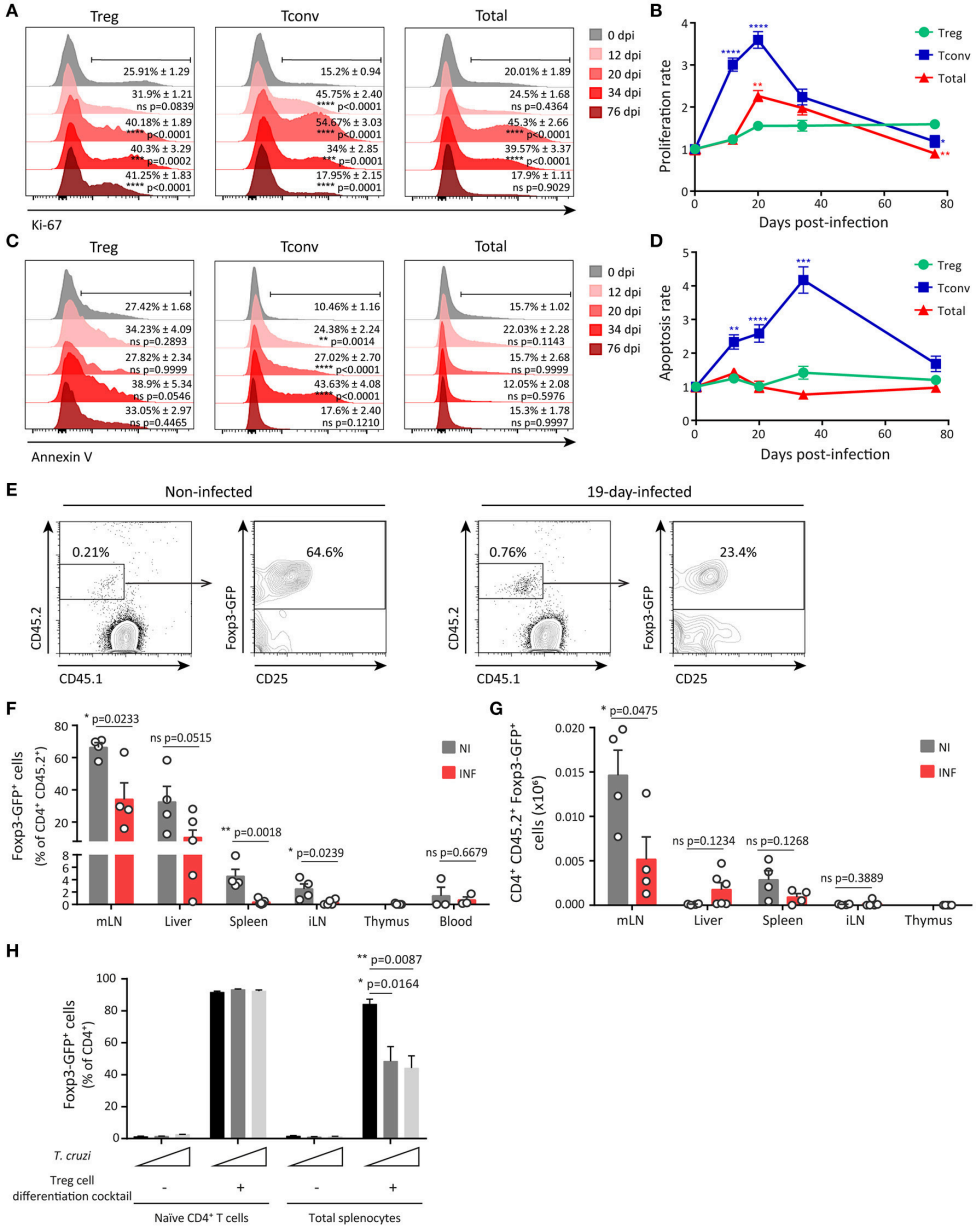
Limited expansion and reduced peripheral differentiation of Treg cells during acute *T. cruzi* infection. **(A)** Histograms for Ki-67 expression in Treg (CD4^+^ Foxp3^+^), Tconv (CD4^+^ Foxp3^−^) and total cells from the spleen of Foxp3-EGFP mice infected with *T. cruzi* at different dpi. Percentage of positive cells mean ± SEM (*n* = 3–6 animals) and *p*-values are indicated for each dpi in comparison to 0 dpi. **(B)** Proliferation rate of cell populations at different dpi calculated as the relation between % of Ki-67^+^ cells at 0 dpi and the other time points according to **(A)**. **(C)** Histograms for Annexin V staining in 7-AAD^−^ gated Treg, Tconv and total cells from the spleen of *T. cruzi* infected Foxp3-EGFP mice at different dpi. Percentage of positive cells mean ± SEM (*n* = 3–6 animals) and *p*-values are indicated for each dpi. **(D)** Apoptosis rate of cell populations at different dpi calculated as the relation between % of Annexin V^+^ cells at 0 dpi and the other time points according to **(C)**. In **(B,D)** statistics were performed by One Way ANOVA comparing Tconv and total cells rates to Treg cells rate at each dpi (Sidak's multiple comparisons test). ^*^P ≤ 0.05, ^**^P ≤ 0.01, ^***^P ≤ 0.001, ^****^P ≤ 0.0001 and *ns* = not significant. **(E–H)** Tconv cells (CD4^+^ CD25^−^ Foxp3-GFP^−^) from non-infected Foxp3-EGFP CD45.2 donors were transferred into CD45.1 recipient mice, which were simultaneously infected with *T. cruzi*. Nineteen days after transfer, conversion of transferred cells (GFP-) into Treg cells (GFP^+^) was assessed in different organs. Representative dot plots showing donor and host CD4^+^ cells according to CD45.2 and CD45.1 staining and Foxp3-GFP expression in CD45.2^+^ CD4^+^ donor cells in mLN from non-infected (NI) or infected (INF) hosts **(E)**. Bar graphs depicting percentage **(F)** and absolute numbers **(G)** of Treg cells within the population of transferred cells in different organs. Each circle represents one animal. Data were pooled from two independent experiments. *P*-values were calculated by unpaired *t*-test. **(H)** Frequency of Treg cells (GFP^+^) determined within the CD4^+^ gate in cultures of purified naïve CD4^+^ T cells or total splenocytes stimulated with coated anti-CD3 and anti-CD28 in the presence or absence of the Treg cell differentiation cocktail and live parasites (at a cell:parasite ratio of 1:1 and 2:1) for 3 days. Data were pooled from two independent experiments with two technical replicates. *P*-values were calculated by unpaired *t*-test.

We first evaluated the proliferation of splenic Treg cells, Tconv and total leukocytes along *T. cruzi* infection by determining *ex vivo* the expression of the proliferation marker Ki-67. As depicted in the histograms in Figure 3A, the frequency of Treg cells expressing Ki-67 showed a moderate increase around 20–34 dpi that persisted at least until 76 dpi. In contrast, the frequencies of Ki-67^+^ Tconv and total cells were markedly augmented along the infection with a peak at 20 dpi, returning to the level of non-infected mice by 76 dpi. Thus, Treg cells showed a significantly lower proliferation rate than Tconv and total cells (Figure 3B). These differences in proliferation may account for the reduced frequency of Treg cells during the early acute phase of *T. cruzi* infection. Next, we evaluated whether differential cell death within each cell subset may also be involved in Treg cell frequency reduction during *T. cruzi* infection. Determination of the frequency of apoptotic cells by Annexin V and 7-AAD staining, and calculation of the apoptosis rate, revealed that *T. cruzi* infection increased cell death particularly within the Tconv population (Figures 3C,D), as previously reported (58). Therefore, an increased cell death within Treg cells was ruled out as a mechanism underlying the decrease in the frequency of this cell subset during *T. cruzi* infection.

We next investigated whether infection with *T. cruzi* restrained Treg cell development and consequently, reduced Treg cell frequency in the periphery. Considering that the numbers of Treg cells are conserved in the thymus of infected mice (Supplementary Figure 1C), we speculated that thymic Treg cell development was not significantly altered during *T. cruzi* infection. Then, we focused on the development of peripheral Treg (pTreg) cells through an *in vivo* approach in order to evidence alterations triggered by *T. cruzi* infection. To this end, CD25^−^ GFP^−^ CD4^+^ cells were purified from the spleen of non-infected CD45.2^+^ Foxp3-GFP reporter mice and injected into CD45.1 WT hosts that were immediately infected. Adoptively transferred non-infected hosts were examined in parallel and used as controls. Twenty days after injection, transferred cells were identified by CD45.2 expression within different organs and the frequency of pTreg was determined according to the up-regulation of Foxp3 and CD25 expression. As illustrated in Figure 3E, mLN from non-infected and infected hosts contained a small percentage of injected cells. A high percentage (around 60%) of the injected cells within mLN from non-infected mice consisted of pTreg cells while most of the exogenous cells in mLN from infected mice were Tconv. The significant reduction in the frequency of pTreg cells originated from the injected Tconv during *T. cruzi* infection was observed not only in mLN that have an environment prone for pTreg cell differentiation, but also in the spleen and iLN (Figure 3F). To confirm that induction of pTreg cells was inhibited during *T. cruzi* infection, we calculated the absolute numbers of newly differentiated pTreg cells in the different organs. In agreement with the frequency data, the highest absolute numbers of induced pTreg cells were found in mLN in comparison to other organs (Figure 3G). Of note, the numbers of pTreg cells were significantly reduced in mLN from infected mice in comparison to non-infected controls but not in other organs. These data further support the notion that differentiation of pTreg cells is reduced during *T. cruzi* infection.

In order to identify mediators involved in the relative decrease of Treg cells during *T. cruzi* infection, we focused on particular inflammatory cytokines that are increased in the course of this parasitic infection and/or have been reported to modulate peripheral Treg cell proliferation and differentiation. Among these mediators, we selected cytokines, such as IL-6 and IL-1β that are produced during *T. cruzi* infection [Supplementary Figure 2C and references (59, 60)] and have been reported to restrain pTreg cell development by favoring a Th17 fate (61, 62). Also, type I interferons increase early during *T. cruzi* infection (63) and have been shown to limit regulatory responses by reducing Treg cell proliferation (26) and peripheral induction (64) in viral infection settings. We determined that mice with deficient production of IL-6 due to deletion of the *Il6* gene (*Il6*^−/−^) or reduced IL-1β/IL-18 due to the lack of Caspase1/11 (*Casp1/11*^−/−^) exhibited a similar decrease in Treg cell frequency at 20 dpi when compared to WT mice. Similarly, infected mice deficient in IFNAR, the specific receptor for type I IFNs, also showed a relative reduction of Treg cell responses (Supplementary Figure 3A). These results highlight that IL-6, IL-1β, and type I IFN signaling were not responsible of the relative Treg cell reduction triggered by *T. cruzi* infection.

As the frequency of Treg cells inversely correlated with parasitemia (Figure 2B), we next speculated that parasites could influence the size of the Treg cell pool during *T. cruzi* infection. To gain further insights in this direction, we evaluated whether changes in parasite levels as consequence of inoculation with different parasite doses have any impact in the frequency of Treg cells in periphery. We determined that 10-fold changes in the parasite dose used for infection resulted in significant dose-dependent differences in parasitemia at 11 dpi but not at 20 dpi, when parasitemia reached a maximum that was independent on the initial infective dose (Supplementary Figure 3B). Of note, the frequency of splenic Treg cells at 11 dpi was inversely associated with parasitemia and therefore remained unchanged in mice infected with the lowest dose, that showed barely detectable parasites in blood, but were significantly and dose-dependently reduced in mice infected with the intermediate and highest infective doses. These differences in Treg cell frequency were no longer observed at 20 dpi, when all mouse groups showed the same parasitemia (Supplementary Figure 3C). In this context, we designed *in vitro* experiments aimed to evaluate whether *T. cruzi* parasites are capable of inhibiting pTreg cell induction. As shown in Figure 3H, most naïve CD4^+^ T cells differentiated into iTreg cells when activated in the presence of a Treg cell differentiation cocktail containing TFG-β, IL-2, and *all trans* retinoic acid (atRA). Of note, live trypomastigotes were unable to inhibit this differentiation. Similarly, more than 90% of the CD4^+^ T cells became iTreg cells when total splenocytes were activated in the presence of the Treg cell differentiation cocktail. In contrast to the lack of effect on purified CD4^+^ T cells, parasites significantly inhibited induction of iTreg cells in cultures of total splenocytes. These results strongly suggest that *T. cruzi* itself is able to actively inhibit Treg cell induction by an indirect mechanism that may depend on accessory cells present within the splenocytes rather than by a direct effect on naïve CD4^+^ T cells.

### Phenotypical and transcriptional characterization of treg cells during *T. cruzi* infection underscore a specialization in the regulation of type 1 effector responses

**Figure 4:**
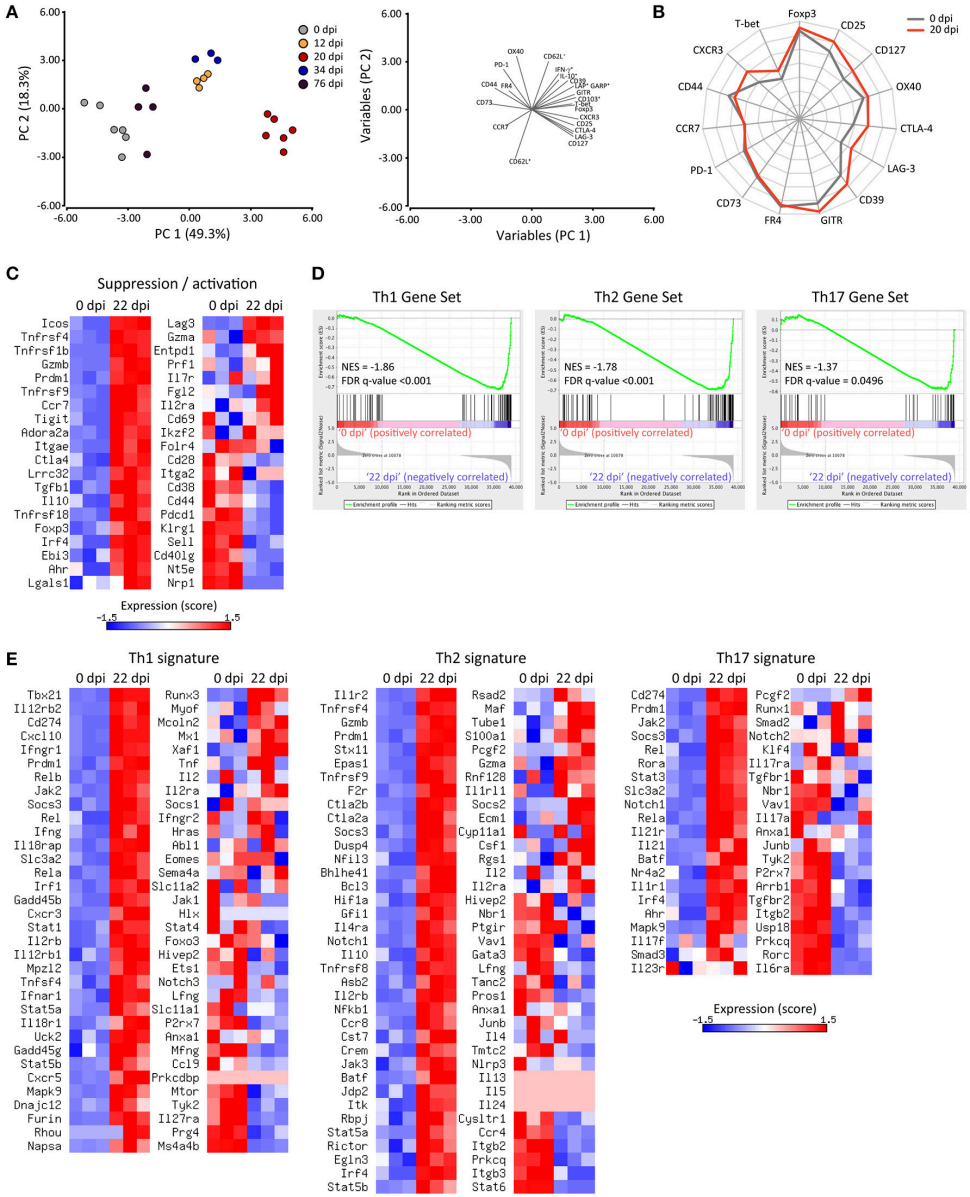
Treg cells show an activated phenotype and acquire a Th1-like profile during *T. cruzi* infection. **(A)** Plots of principal component analysis (PCA) for surface and intracellular markers of Treg cells from the spleens of Foxp3-EGFP mice at the indicated dpi (left panel) and the variables included in PC1 and PC2 (right panel). Each circle represents one animal. **(B)** Star plot displaying the expression of the indicated markers in Treg cells from non-infected (0 dpi, gray line) or infected (20 dpi, red line) mice. Each spoke of the star represents the geometric mean of fluorescence intensity for the indicated marker in a log2 scale from 1 to 16,384. The data length of a spoke is proportional to the MFI of expression of that marker in the corresponding sample. **(C)** Heat maps for the normalized expression scores (row mean values of 0 and variance of 1) of genes encoding suppression and/or activation markers in Treg cells (CD4^+^ CD25^+^ Foxp3-GFP^+^) purified from the spleen of non-infected (0 dpi) or 22-days-infected (22 dpi) Foxp3-EGFP mice. **(D)** Gene set enrichment analysis of the Treg cell transcriptome for Th1, Th2 and Th17 signatures using selected gene sets. **(E)** Heat maps for the normalized expression scores (row mean values of 0 and variance of 1) of the selected genes used in **(D)**. RNAseq data are from one experiment with three biological replicates. The PCA plot, star plot and heat maps were created using InfoStat software, Microsoft Excel spreadsheet and Matrix2png online tool, respectively.

The relative deficiency in the magnitude of Treg cell responses during the course of *T. cruzi* infection may be compensated by the activation and enhancement of the immunosuppressive function of this subset as reported previously (65–67). To address this, we performed a comprehensive evaluation of Treg cell phenotype by flow cytometry, determining the expression of proteins associated with Treg cell activation and suppressive function, such as CD25, CTLA-4, GITR, CD39, CD73, LAG-3, OX40, PD-1, FR4, GARP, TGF-β, and IL-10 together with molecules involved in migration and effector/memory subset classification like CD103, CD127, CCR7, CD44, and CD62L. We also assessed markers of Th1 specialization including T-bet, CXCR3 and IFN-γ in these cells. In order to broadly interpret the phenotypic changes occurring in Treg cells during the progression of *T. cruzi* infection, we focused on changes occuring in spleen and performed multivariate analysis of all the markers evaluated. Principal component analysis (PCA) defined two principal components (PC) that accounted for around 70% of the total variance and allowed to evidence the phenotypic changes suffered by Treg cells along the different dpi (Figure 4A, left pannel). The variables included in PC1 and PC2 as well as the direction of their changes are also depicted (Figure 4A, right pannel). Interpretation of the PCA plots indicated that the phenotype of Treg cells changed progressively from 0 dpi (non-infected) until 20 dpi (maximal difference) by modifications in the variables included mainly in PC1 and at a lower extent in PC2. This analysis also established that Treg cells at 12 and 34 dpi showed a similar phenotype that is intermediate between those of cells from 0 and 20 dpi while Treg cells at 76 dpi showed features more related to the phenotype of Treg cells from non-infected mice (0 dpi).

The kinetics of expression of each marker highlighted that Treg cells from *T. cruzi* infected mice exhibited phenotypic changes as early as 12 dpi, with a maximum between 20 and 34 dpi (Supplementary Figure 4A). Of note, Treg cells from 20-days infected mice showed an evident activated phenotype as demonstrated by the upregulation of activation and memory markers, such as Foxp3, CD25, OX40, CD103, and CD127 in comparison to Treg cells from non-infected mice (68). In addition, Treg cells at 20 dpi showed increased expression of molecules associated with their suppressive function including CTLA-4, GITR, CD39, and LAG-3 but not CD73, PD-1, and FR4. We also established that Treg cells increased their capacity to produce regulatory cytokines, such as TGF-β (as indicated by co-expression of GARP and LAP) and IL-10 (Supplementary Figure 4B) after 20 days of infection. Finally, we determined that Treg cells at 20 dpi upregulated CXCR3 and slightly T-bet, and increased IFN-γ production in comparison to counterparts from non-infected mice (0 dpi; Supplementary Figures 4C,D). Remarkably, T-bet and CXCR3 expression levels in Treg cells from 20 dpi were comparable and higher than those observed in Tconv cells at the same time point, respectively (Supplementary Figure 4C). The expression of several markers studied were represented in a star plot that allowed to evidence differences in the phenotypic profile of Treg cells at 0 and 20 dpi, showing that *T. cruzi* infection induced the up-regulation of activation and Th1-associated markers as well as immunosuppressive mediators (Figure 4B).

In order to determine the global impact of *T. cruzi* infection on the transcriptional program of Treg cells, we compared by RNAseq the gene expression profiles of Treg cells purified from the spleen at 0 and 20 dpi. A total of 5,175 genes were differentially expressed in Treg cells as a consequence of *T. cruzi* infection (data not shown). As illustrated by the heat maps in Figure 4C, Treg cells from infected mice exhibited a global increase in the levels of many transcripts encoding activation markers and suppressive mediators. In addition to those noticed by flow cytometry (CD25, CD127, CD103, GITR, CTLA-4, TGF-β, and IL-10 among others), we also detected an up-regulation of genes encoding ICOS, Granzyme B, CD137, TIGIT, Adenosin A2a receptor, EBI3, Galectin-1, among others. In agreement with their phenotypic profile, Treg cells from infected mice showed reduced amounts of transcripts encoding CD44, PD-1, CD62L, and CD73. Of note, Treg cells from infected mice showed augmented levels in the expression of Blimp-1 and IRF4, transcription factors that drive effector Treg cell differentiation (67). Altogether, this transcriptional profile reflected a clear activation of Treg cells along *T. cruzi* infection. Next, we aimed to determine whether Treg cells from infected mice exhibit programs that denote any functional specialization (69). To this end, we performed Gene Set Enrichment Analysis of the Treg cell transcriptome for Th1, Th2, and Th17 signatures. As shown in the enrichment plots, the transcriptome of Treg cells from infected mice are significantly enriched in the Th1, Th2, and Th17 gene sets (Figure 4D). Interestingly, Treg cells from *T. cruzi* infected mice up-regulated most of the genes classically associated with a Th1 specialization including *Tbx21, Ifng*, and *Cxcr3* and also the two subunits of the IL-12 receptor (*Il12rb2* and *Il12rb1*), *Stat1, Ifngr1*, and *Cxcl10* while they down-regulated *Il27ra* (Figure 4E). Of note, Treg cells from infected mice showed increased levels of some transcripts associated with a Th2 signature, such as *Il1r2, Il4ra, Il10*, and *Irf4* but not of other prototypical Th2 genes, such as *Gata3, Il4, Il5*, or *Il13*. Similarly, this cell subset showed increased expression of certain genes associated with a Th17 fate like *Rora, Stat3, Il21r*, and *Ahr* but not of others tightly linked to Th17 cells like *Rorc* and *Il17a*.

### Adoptive transfer of treg cells suppresses anti-parasite effector response and diminishes parasite control during *T. cruzi* infection

Once established the features acquired by Treg cells after *T. cruzi* infection, we aimed at evaluating the biological role of this cell subset in the progression of the infection. Considering the results from Figure 2, we were particularly interested in addressing the relevance of the limited Treg cell response for the induction/maintenance of effector responses and immunopathology. To this end, we designed an experiment in which Treg cell numbers were increased by the injection of Treg cells differentiated *in vitro* (iTreg cells). iTreg cells were obtained by sorting Foxp3-GFP^+^ cells from cultures in which CD4^+^ T cells obtained from Foxp3-EGFP reporter mice were stimulated with anti-CD3 and anti-CD28 in the presence of the Treg cell differentiation cocktail (Supplementary Figure 5A). In agreement with data showing that this cocktail induced stable human iTreg cells (70), we determined that iTreg cells generated in these conditions showed immunosuppressive capacity *in vitro* (Supplementary Figure 5B), and failed to produce effector cytokines, such as IFN-γ and IL-17 (Supplementary Figure 5C).

**Figure 5:**
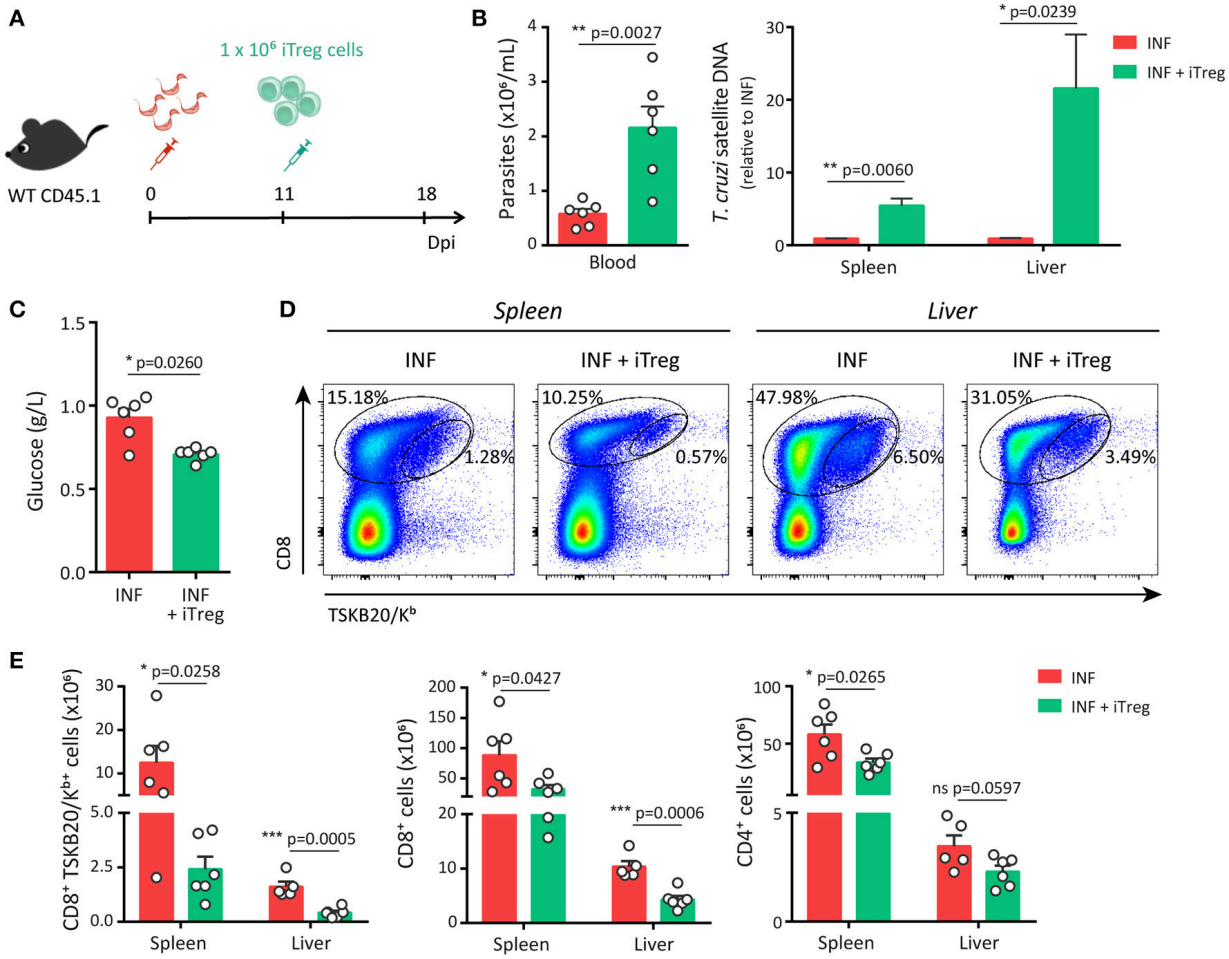
Adoptive transfer of Treg cells restricts the development of a robust parasite-specific CD8^+^ T cell response during acute *T. cruzi* infection. **(A)** CD45.1 WT mice were infected with *T. cruzi* and 11 days later were transferred with 1 × 10^6^ iTreg cells differentiated from Foxp3-EGFP mice. **(B–C)** Parasitemia [**(B)**, left], parasite load by real time PCR in the spleen and liver [**(B)**, right] and glucose concentration in the plasma **(C)** of non-transferred control (INF) and transferred (INF + iTreg) infected mice. **(D–F)** Dot plots **(D)** and graphs showing absolute numbers **(E)** of total and parasite-specific T cells responses in the spleen and liver of transferred and control mice. Data are presented as mean + SEM and are pooled from two independent experiments. *P*-values were calculated by unpaired *t*-test **(B,E)** or Mann Whitney test **(C)**.

As schematized in Figure 5A, adoptive transfer experiments were designed to inject iTreg cells into infected hosts at 11 dpi, the time point in which the reduction of Treg cell frequency becomes significant. Remarkably, infected hosts that received iTreg cells showed significantly augmented levels of parasites in blood and tissues in comparison to control counterparts at 18 dpi (Figure 5B). In addition, and likely as consequence of the uncontrolled parasite replication, mice injected with iTreg cells presented reduced blood glucose concentration but biochemical markers of tissue damage were not affected (Figure 5C and data not shown). In order to establish the causes of the reduced parasite control, we evaluated the magnitude of the effector immune response. Adoptive transfer of iTreg cells significantly diminished the frequency of parasite-specific CD8^+^ T cells in spleen and liver of infected mice (Figure 5D). Furthermore, the absolute numbers of parasite-specific CD8^+^ T cells and total CD8^+^ and CD4^+^ T cells were also reduced after the injection of iTreg cells in infected hosts (Figure 5E). Altogether, these results indicate that increasing Treg cell numbers during *T. cruzi* infection severely compromises the magnitude of protective immune responses including parasite-specific CD8^+^ T cell immunity and consequently, limits the control of parasite replication and host resistance to the infection.

## Discussion

The role played by Treg cells in the progression of *T. cruzi* infection remains controversial likely because any rational mechanistic evaluation is precluded by the limited characterization of the triggered Treg cell response. Considering this, we studied in detail the kinetics of Treg cell responses using Foxp3-EGFP reporter mice and demonstrated that frequency of Treg cells is reduced along *T. cruzi* infection in most peripheral organs including spleen, liver and inguinal lymph nodes. Furthermore, we showed that this relative decrease is not caused by a reduction in Treg cell absolute numbers but rather by a significant expansion of effector cells that affect the ratio of Treg cells to other immune subsets in different tissues. Thus, our results further expand and complement a recent work reporting that frequency of splenic Treg cells is significantly reduced during acute experimental *T. cruzi* infection likely as result of a neuroendocrine dysbalance (41). Altogether, this report and our data underscore that, differently from most chronic infections of viral, bacterial and parasitic origin which are characterized by a Treg cell accrual, *T. cruzi* infection associates with a significant decrease in the magnitude of the Treg cell response that is prolonged even until the early chronic phase of the infection.

After evaluating recognized mechanisms that regulate Treg cell pool size in periphery, we established that Treg cells exhibited significantly lower proliferation rate in comparison to effector immune subsets during *T. cruzi* infection, confirming a previous report in this direction (41). Although not surprising considering the current views on pTreg cell induction (71), we also demonstrated that the inflammatory environment triggered by *T. cruzi* limits the peripheral induction of Treg cells. Remarkable, infections characterized by Treg cell accumulation triggered a marked proliferation of Treg cells but not peripheral induction of this cell subset. Thus, influenza infection was shown to induce peripheral proliferation and accumulation of Ag-specific thymically derived Treg cells whereas conventional CD4^+^ T cells with identical specificity for the pathogen underwent little or no peripheral conversion in infected mice (72). Similarly, Treg cells expressing a pathogen-specific transgenic TCR were found to expand in response to *M. tuberculosis* without *de novo* induction of pTreg cells (73). Of note, proliferation of Ag-specific Treg cells was detected during *M. tuberculosis* but not *L. monocytogenes* infection indicating that only certain microbes are able to induce an inflammatory milieu that is conducive to the expansion of Ag-specific Treg cells (74). In this context, the results presented in our manuscript support the notion that *T. cruzi* infection triggers a relatively deficient Treg cell response by inducing an inflammatory context that does not support pTreg cell induction (somehow expected as discussed) but also limits Treg cell proliferation. Given that Treg cells have a high dependence on exogenous IL-2 for differentiation and proliferation, it is likely that Treg cell homeostasis may be affected by the suppressed production or increased consumption of IL-2 that occurs during *T. cruzi* infection (75–77). Indeed, IL-2 consumption by proliferating effector cells resulted in reduced Treg numbers in infections with *Toxoplasma gondii, Listeria monocytogenes*, and vaccinia virus (25). In this regard, we demonstrated that the systemic concentration of IL-2 remained unchanged during the acute phase of *T. cruzi* infection and that it showed no correlation with the frequency of splenic Treg cells. In addition, Gonzalez et al (41) reported that treatment with recombinant IL-2 was not sufficient to increase Treg cell frequency during this parasitic infection. These data suggest that regulation of the Treg cell pool size during *T. cruzi* infection is independent from IL-2 levels.

Several pro-inflammatory mediators have been reported to modulate Treg cell homeostasis during inflammatory conditions (78). Among them, IL-1β and IL-6 emerged as possible candidates to mediate the limited Treg cell response during *T. cruzi* infection, given their largely known function to promote a Th17 over a Treg cell fate (61, 62). We previously demonstrated that Th17 and other IL-17^+^ cell subsets are induced early during *T. cruzi* infection (79, 80) and when compared, the kinetics of the increment in Th17 cell frequencies and the relative decrease of Treg cells showed a reciprocal behavior (data not shown). Also, type I IFNs may be involved in the limited proliferation and induction of Treg cells as reported in viral infections (26, 64). However, evaluation of the magnitude of Treg cell responses in infected mice that lacked IL-6, IL-1β, and type I IFNs signaling due to genetic deletions in *Il6, Caspase1/11*, and *Ifnar* genes, respectively, ruled out a main role for these pro-inflammatory cytokines in the regulation of the Treg cell size during *T. cruzi* infection. Considering other possible inflammatory mediators involved in this phenomenon; we established that frequency of Treg cells showed a strong inverse correlation with the levels of parasite in blood along the infection. Furthermore, *in vitro* experiments indicated that live parasites are able to inhibit the induction of Treg cells by a mechanism that depends on cell populations other than CD4^+^ T cells themselves. Altogether, these findings strongly suggest that the *T. cruzi* load affects Treg cell peripheral induction and/or proliferation by indirect mechanisms that are currently under investigation in our laboratory.

Previous studies aimed at addressing the biological relevance of Treg cells in the course of *T. cruzi* infection reported contradictory results regarding the role of this subset in the regulation of the magnitude and functionality of effector immune responses, parasite control and development of immunopathology. Thus, Treg cell depletion by treatment with anti-CD25 depleting antibodies was reported to have a limited role in the induction of parasite-specific CD8^+^ T cells, and therefore, parasite control and host survival (43), but also to slightly increase host resistance to infection by favoring activation of CD4^+^ T cells (44). In contrast, combined injection of anti-CD25 and anti-GITR evidenced that Treg cells may be important to prevent exuberant inflammation, particularly at the heart, and increased mortality during this parasitic infection (42). More recently, early and sustained Treg cell depletion with anti-CD25 was reported to modulate Th1 and Th17 responses during *T. cruzi* infection, reducing cardiac parasitosis and inflammation (45). The reasons underlying these controversial data have not yet been determined but may be related to the variability in parasite and host strains, timing of Treg manipulation and the use of non-specific Treg cell depletion strategies. Considering these data and our results demonstrating a limited Treg cell response during *T. cruzi* infection, we reasoned that increasing Treg cell numbers may be a more rational strategy to define the role of Treg cells in this infection setting. Accordingly, we designed adoptive transfer experiments in which Treg cell numbers were manipulated by injection of *in vitro* differentiated Treg cells. This approach has been widely used to modulate the outcome of several pathological conditions including autoimmunity and GVHD (81). We determined that increasing Treg cells at the time when infected mice exhibited a significant reduction in the frequency of this cell subset, limited the accumulation of parasite-specific CD8^+^ T cells, severely compromising the control of parasite replication in tissues and host resistance to *T. cruzi*. These data are significant though they should be interpreted with caution as the activation status of *in vitro* generated Treg cells may enhance their suppressive function in comparison to endogenous Treg cells as previously reported (82). Even considering these limitations, our findings demonstrate that activated Treg cells are able to dampen specific CD8^+^ T cell immunity during *T. cruzi* infection. Furthermore, these data suggest that the natural contraction of Treg cell responses observed during the acute phase of *T. cruzi* infection may be critical to allow the emergence of a robust effector response aimed at controlling pathogen replication as previously reported for acute infections (25). Thus, timing and other features of the Treg cell response during this parasitic infection may evidence a particular mechanism of a chronic *T. cruzi*-host adaptation that allows the emergence of effector responses able to sustain partial parasite control and host resistance but preventing complete pathogen elimination. Within this conceptual framework, further research will be required to address the mechanisms involved and to determine if a precise Treg cell manipulation may represent an opportunity to potentiate anti-parasite immunity and parasite control.

It is currently known that Treg cells exert their regulatory function by deploying a plethora of immunosuppressive mechanisms that target different immune cell populations. Treg cells have been reported to modulate the activation, proliferation and/or function of CD8^+^ and CD4^+^ T cells, B cells, NK cells, monocytes and dendritic cells, among others (1). Altogether, the phenotypic, transcriptional and functional profiles of Treg cells activated during *T. cruzi* infection highlight the global activation of this cell subset and also evidence a marked heterogeneity in the Treg cell compartment from infected mice. Of note, even though GSEA suggests that *T. cruzi* infection elicit Treg cell subsets that acquire specialized programs for the regulation of Th1, Th2, and Th17 responses, these cells showed a global up-regulation of most of the genes classically associated to the Th1 signature but not of those critical for Th2 and Th17 fates, such as Gata-3 and Rorγt. Therefore, this specialized program may tailor Treg cells with a particular ability to suppress *in vivo* type 1 effector responses guiding Treg cells to Th1 inflammatory sites. Further studies including single-cell RNA-seq may be very helpful to precisely unravel the complexities of Treg cell responses during this infection. By potentiating the regulatory response through iTreg cell adoptive transfer, we established that Treg cells activated in the context of *T. cruzi* infection have the ability to suppress total and parasite-specific CD8^+^ T cell immunity and, at a minor extent, the polyclonal CD4^+^ T cell response. Whether Treg cells also influence other immune cell populations in these infectious setting remains unexplored. Although not previously reported for *T. cruzi* infection, Treg cell mediated suppression of CD8^+^ T cell immunity has been widely described during infections, particularly of viral origin (13). Treg cells are able to regulate many steps of the CD8^+^ T cell response through mechanisms that remain partially elucidated and involve not only the CD8^+^ T cells themselves but also antigen presenting cells. In this way, antigen-specific and polyclonal Treg cells impaired CD8^+^ T cell priming by inhibiting the early expansion of antigen-specific cells (83) and preventing the activation of antigen-presenting cells through CTLA-4 mediated inhibitory signals (84). Even more, Treg cells have been reported to directly limit CD8^+^ T cell proliferation (85), differentiation into effector cells (86), cytotoxic effector function (87, 88) and to sustain CD8^+^ T cell exhaustion (89) by mechanisms that involve IL-2 consumption, TGF-β and IL-10 production, PD-1 and CD39 expression, and many others. According to this, Treg cells activated in the context of *T. cruzi* infection acquired a phenotypic profile that would allow direct and indirect regulation of CD8^+^ T cell immunity. In this regard, a direct suppressive function is supported by the increased expression of CXCR3 in Treg cells activated during *T. cruzi* infection that would allow migration to inflammatory sites. In addition, a direct suppressive mechanism may be responsible for the reduction in the magnitude of the specific CD8^+^ T cell immunity that occur following iTreg cell adoptive transfer at 11 dpi, a time point after the emergence of the parasite-specific CD8^+^ T cell response. On the other hand, the fact that Treg cells activated during *T. cruzi* infection expressed remarkably high levels of CTLA-4 may indicate that these cells are particularly prepared for *in vivo* regulation of antigen-presenting cells. A detailed comprehension of the mechanisms underlying the Treg cell-mediated inhibition of CD8^+^ T cell immunity during this parasitic infection will be essential to design possible immune-intervention strategies aimed at improving parasite-specific immunity without enhancing infection-associated pathology.

Altogether, our data delineate a model in which infection with *T. cruzi* promotes activation but limits proliferation and peripheral induction of Treg cells during the acute phase. Whether these events are mediated by active mechanisms remains to be clearly established as passive pathways are not completely ruled out by our study. In any case, the Treg cell mediated regulatory response seems to exhibit a putatively improved quality but reduced quantity during *T. cruzi* infection. This weakened Treg cell response allows the emergence of a robust parasite-specific effector immunity, particularly of CD8^+^ T cells, which partially controls parasite replication favoring host resistance. Although these findings support a deleterious role for Treg cells during the acute phase of *T. cruzi* infection, a special attention needs to be given to timing. Indeed, we also determined that during the chronic phase, when parasite replication is limited and inflammation goes down, the frequency and phenotypic profile of Treg cells tended to return to normal conditions. This finding together with reported data in which increased Treg cell frequency and function in chagasic patients correlate with better clinical outcomes, likely as consequence of a limited chronic inflammation, proposes that Treg cell role may switch during *T. cruzi* infection from deleterious in the acute phase to protective during chronic Chagas disease. Further research will be required to definitively address this point in order to establish a rational framework for the design of novel treatment strategies to differentially manipulate Treg cells during different stages of *T. cruzi* infection.

## Ethics statement

This study was carried out in accordance with the recommendations of Guide to the care and use of experimental animals (Canadian Council on Animal Care, 1993) and Institutional Animal Care and Use Committee Guidebook (ARENA/OLAW IACUC Guidebook, National Institutes of Health, 2002). The protocol was approved by the Institutional Animal Care and Use Committee (IACUC) Facultad de Ciencias Químicas, Universidad Nacional de Córdoba (Approval Number 565/15 and 731/18) (OLAW Assurance number F16-00193-A5802-01).

## Author contributions

CA designed and performed most of the experiments, analyzed data, and wrote/commented on the manuscript. JT, CR, FC, FF, SB, and CB performed experiments and commented on the manuscript. VA and OJ participated in the execution and analysis of the RNAseq experiment and provided funding [Fondation pour la Recherche Médicale (AJE201212 to OJ), the Région Midi-Pyrénées (OJ)]. CM and AG participated in data analysis, commented on the manuscript and provided funding. EA supervised the research, designed experiments, wrote the manuscript, and provided funding.

### Conflict of interest statement

The authors declare that the research was conducted in the absence of any commercial or financial relationships that could be construed as a potential conflict of interest. The reviewer DA and handling editor declared their shared affiliation at time of review.
