# Supplementary material for: Limited Foxp3+ Regulatory T Cells Response During Acute Trypanosoma cruzi Infection Is Required to Allow the Emergence of Robust Parasite-Specific CD8+ T Cell Immunity
Source: Front Immunol. 2018 Nov 5;9:2555. doi: 10.3389/fimmu.2018.02555 (PMC6230662; doi:10.3389/fimmu.2018.02555)
Supplement: Supplementary file 1 [file Data_Sheet_1.PDF]

Supplementary Table 1. List of genes selected for Th1, Th2 and Th17 gene sets (signature) and the corresponding reference

Gene set (signature)	Gene name	Reference	Pubmed id
Th1	Abl1	Chen A et al., Mol Cell Biol 2014	https://www.ncbi.nlm.nih.gov/pubmed/21690296
Th1	Anxa1	Gavins FN et al., Front Immunol 2012	https://www.ncbi.nlm.nih.gov/pubmed/23230437
Th1	Ccl9	Lin CC et al., Nat Commun 2014	https://www.ncbi.nlm.nih.gov/pubmed/24699451
Th1	Cd274	Loke P et al., Proc Natl Acad Sci 2003	https://www.ncbi.nlm.nih.gov/pubmed/12697896
Th1	Cxcl10	Ranzani V et al., Nat Immunol 2015	https://www.ncbi.nlm.nih.gov/pubmed/25621826
Th1	Cxcr3	Sallusto F et al., Immunol Today 1998	https://www.ncbi.nlm.nih.gov/pubmed/9864948
Th1	Cxcr5	Sallusto F et al., Immunol Today 1998	https://www.ncbi.nlm.nih.gov/pubmed/9864948
Th1	Dnajc12	Ranzani V et al., Nat Immunol 2015	https://www.ncbi.nlm.nih.gov/pubmed/25621826
Th1	Eomes	TYang Y et al., J Immunol 2008	https://www.ncbi.nlm.nih.gov/pubmed/19050290
Th1	Ets1	Grenningloh R et al., J Exp Med 2005	https://www.ncbi.nlm.nih.gov/pubmed/15728239
Th1	Foxo3	Stienne C et al., Immunity 2016	https://www.ncbi.nlm.nih.gov/pubmed/27742544
Th1	Furin	Pesu M et al., Blood 2006	https://www.ncbi.nlm.nih.gov/pubmed/16627761
Th1	Gadd45b	Chi H et al., EMBO J 2004	https://www.ncbi.nlm.nih.gov/pubmed/15044949
Th1	Gadd45g	Lu B et al., Immunity 2001	https://www.ncbi.nlm.nih.gov/pubmed/11371360
Th1	Hivep2	Nakayama T et al., Adv Exp Med Biol 2010	https://www.ncbi.nlm.nih.gov/pubmed/20795536
Th1	Hlx	Zheng WP et al., J Immunol 2004	https://www.ncbi.nlm.nih.gov/pubmed/14688316
Th1	Hras	Iborra S et al., Blood 2011	https://www.ncbi.nlm.nih.gov/pubmed/21444916
Th1	Ifnar1	Nguyen KB et al., Science 2002	https://www.ncbi.nlm.nih.gov/pubmed/12242445
Th1	Ifng	Mosmann TR et al., J Immunol. 1986	https://www.ncbi.nlm.nih.gov/pubmed/2419430
Th1	Ifngr1	Szabo SJ et al., Annu Rev Immunol 2003	https://www.ncbi.nlm.nih.gov/pubmed/12500979
Th1	Ifngr2	Szabo SJ et al., Annu Rev Immunol 2003	https://www.ncbi.nlm.nih.gov/pubmed/12500979
Th1	Il12rb1	Hsieh CS et al., Science 1993	https://www.ncbi.nlm.nih.gov/pubmed/8097338
Th1	Il12rb2	Szabo SJ et al., J Exp Med 2002	https://www.ncbi.nlm.nih.gov/pubmed/9120387
Th1	Il18r1	Srinivasan A et al., J Immunol 2007	https://www.ncbi.nlm.nih.gov/pubmed/17475863
Th1	Il18rap	Srinivasan A et al., J Immunol 2007	https://www.ncbi.nlm.nih.gov/pubmed/17475863
Th1	Il2	Owen DL et al., F1000Res 2017	https://www.ncbi.nlm.nih.gov/pubmed/28163905
Th1	Il27ra	Cox JH et al., J Exp Med 2011	https://www.ncbi.nlm.nih.gov/pubmed/21173106
Th1	Il2ra	Owen DL et al., F1000Res 2017	https://www.ncbi.nlm.nih.gov/pubmed/28163905
Th1	Il2rb	Owen DL et al., F1000Res 2017	https://www.ncbi.nlm.nih.gov/pubmed/28163905
Th1	Irf1	Kano S et al., Nat Immunol 2008	https://www.ncbi.nlm.nih.gov/pubmed/18059273
Th1	Jak1	Murray PJ, J Immunol 2007	https://www.ncbi.nlm.nih.gov/pubmed/17312100
Th1	Jak2	Murray PJ, J Immunol 2007	https://www.ncbi.nlm.nih.gov/pubmed/17312100
Th1	Lfng	Gu W et al., Plos One 2012	https://www.ncbi.nlm.nih.gov/pubmed/23071776
Th1	Mapk9	Yang DD et al., Immunity 1998	https://www.ncbi.nlm.nih.gov/pubmed/9806643
Th1	Mcoln2	Ranzani V et al., Nat Immunol 2015	https://www.ncbi.nlm.nih.gov/pubmed/25621826
Th1	Mfng	Gu W et al., Plos One 2012	https://www.ncbi.nlm.nih.gov/pubmed/23071776
Th1	Mpzl2	Lin CC et al., Nat Commun 2014	https://www.ncbi.nlm.nih.gov/pubmed/24699451
Th1	Ms4a4b	Venkataraman C et al., J Immunol 2000	https://www.ncbi.nlm.nih.gov/pubmed/10878334
Th1	Mtor	Delgoffe GM et al., Immunity 2009	https://www.ncbi.nlm.nih.gov/pubmed/19538929
Th1	Mx1	Ranzani V et al., Nat Immunol 2015	https://www.ncbi.nlm.nih.gov/pubmed/25621826
Th1	Myof	Ranzani V et al., Nat Immunol 2015	https://www.ncbi.nlm.nih.gov/pubmed/25621826
Th1	Napsa	Ranzani V et al., Nat Immunol 2015	https://www.ncbi.nlm.nih.gov/pubmed/25621826
Th1	Notch3	Jurynczyk M et al., J Immunol 2008	https://www.ncbi.nlm.nih.gov/pubmed/18250475
Th1	P2rx7	Salles EM, Plos Pathog 2017	https://www.ncbi.nlm.nih.gov/pubmed/28859168
Th1	Prdm1	Neumann C et al., J Exp Med 2014	https://www.ncbi.nlm.nih.gov/pubmed/25073792
Th1	Prg4	Ranzani V et al., Nat Immunol 2015	https://www.ncbi.nlm.nih.gov/pubmed/25621826
Th1	Prkcdbp	Ranzani V et al., Nat Immunol 2015	https://www.ncbi.nlm.nih.gov/pubmed/25621826
Th1	Rel	Oh H et al., Immunol Rev 2013	https://www.ncbi.nlm.nih.gov/pubmed/23405894
Th1	Rela	Oh H et al., Immunol Rev 2013	https://www.ncbi.nlm.nih.gov/pubmed/23405894
Th1	Relb	Oh H et al., Immunol Rev 2013	https://www.ncbi.nlm.nih.gov/pubmed/23405894
Th1	Rhou	Ranzani V et al., Nat Immunol 2015	https://www.ncbi.nlm.nih.gov/pubmed/25621826
Th1	Runx3	Djuretic IM et al., Nat Immunol 2007	https://www.ncbi.nlm.nih.gov/pubmed/17195845
Th1	Sema4a	Kumanogoh A et al., Immunity 2005	https://www.ncbi.nlm.nih.gov/pubmed/15780988
Th1	Slc11a1	Maji A et al., Sci Rep 2015	https://www.ncbi.nlm.nih.gov/pubmed/26469538
Th1	Slc11a2	Ranzani V et al., Nat Immunol 2015	https://www.ncbi.nlm.nih.gov/pubmed/25621826
Th1	Slc3a2	Cantor J et al., J Immunol 2011	https://www.ncbi.nlm.nih.gov/pubmed/21670318
Th1	Socs1	Egwuagu CE et al., J Immunol 2002	https://www.ncbi.nlm.nih.gov/pubmed/11907070
Th1	Socs3	Egwuagu CE et al., J Immunol 2002	https://www.ncbi.nlm.nih.gov/pubmed/11907070
Th1	Stat1	Takeada et al., J Immunol 2003	https://www.ncbi.nlm.nih.gov/pubmed/12734330
Th1	Stat4	Kaplan MH et al., Nature 1996	https://www.ncbi.nlm.nih.gov/pubmed/8700209
Th1	Stat5a	Owen DL et al., F1000Res 2017	https://www.ncbi.nlm.nih.gov/pubmed/28163905
Th1	Stat5b	Owen DL et al., F1000Res 2017	https://www.ncbi.nlm.nih.gov/pubmed/28163905
Th1	Tbx21	Szabo SJ et al., Cell 2000	https://www.ncbi.nlm.nih.gov/pubmed/10761931
Th1	Tnf	Romagnani S, Ann Allergy Asthma Immunol 2000	https://www.ncbi.nlm.nih.gov/pubmed/10923599
Th1	Tnfsf4	Croft M et al., Immunol Rev 2009	https://www.ncbi.nlm.nih.gov/pubmed/19426222
Th1	Tyk2	Ishizaki M et al., J Immunol 2011	https://www.ncbi.nlm.nih.gov/pubmed/21606247
Th1	Uck2	Ranzani V et al., Nat Immunol 2015	https://www.ncbi.nlm.nih.gov/pubmed/25621826
Th1	Xaf1	Ranzani V et al., Nat Immunol 2015	https://www.ncbi.nlm.nih.gov/pubmed/25621826
Th2	Anxa1	Gavins FN et al., Front Immunol 2012	https://www.ncbi.nlm.nih.gov/pubmed/23230437
Th2	Asb2	Horiuchi S et al., J Immunol 2011	https://www.ncbi.nlm.nih.gov/pubmed/21536806
Th2	Batf	Betz BC et al., J Exp Med 2010	https://www.ncbi.nlm.nih.gov/pubmed/20421391/
Th2	Bcl3	Corn RA et al., J Immunol 2005	https://www.ncbi.nlm.nih.gov/pubmed/16081776
Th2	Bhlhe41	Yang XO et al., Nat Immunol 2009	https://www.ncbi.nlm.nih.gov/pubmed/19881507
Th2	Ccr4	Morimoto Y et al., J Leukoc Biol 2005	https://www.ncbi.nlm.nih.gov/pubmed/16126843
Th2	Ccr8	Zingoni A et al., J Immunol 1998	https://www.ncbi.nlm.nih.gov/pubmed/9670926

Th2	Crem	Horiuchi S et al., J Immunol 2011	https://www.ncbi.nlm.nih.gov/pubmed/21536806
Th2	Csf1	Lin CC et al., Nat Commun 2014	https://www.ncbi.nlm.nih.gov/pubmed/24699451
Th2	Cst7	Okoye IS et al., Proc Natl Acad Sci	https://www.ncbi.nlm.nih.gov/pubmed/25024218
Th2	Ctla2a	Okoye IS et al., Proc Natl Acad Sci	https://www.ncbi.nlm.nih.gov/pubmed/25024218
Th2	Ctla2b	Okoye IS et al., Proc Natl Acad Sci	https://www.ncbi.nlm.nih.gov/pubmed/25024218
Th2	Cyp11a1	Horiuchi S et al., J Immunol 2011	https://www.ncbi.nlm.nih.gov/pubmed/21536806
Th2	Cysltr1	PArmentier CN et al., J Allergy Clin Immunol 2012	https://www.ncbi.nlm.nih.gov/pubmed/22391114
Th2	Dusp4	Horiuchi S et al., J Immunol 2011	https://www.ncbi.nlm.nih.gov/pubmed/21536806
Th2	Ecm1	Horiuchi S et al., J Immunol 2011	https://www.ncbi.nlm.nih.gov/pubmed/21536806
Th2	Egln3	Okoye IS et al., Proc Natl Acad Sci	https://www.ncbi.nlm.nih.gov/pubmed/25024218
Th2	Epas1	Horiuchi S et al., J Immunol 2011	https://www.ncbi.nlm.nih.gov/pubmed/21536806
Th2	F2r	Horiuchi S et al., J Immunol 2011	https://www.ncbi.nlm.nih.gov/pubmed/21536806
Th2	Gata3	Zheng W et al., Cell 1997	https://www.ncbi.nlm.nih.gov/pubmed/9160750
Th2	Gfi1	Horiuchi S et al., J Immunol 2011	https://www.ncbi.nlm.nih.gov/pubmed/21536806
Th2	Gzma	Horiuchi S et al., J Immunol 2011	https://www.ncbi.nlm.nih.gov/pubmed/21536806
Th2	Gzmb	Devadas S et al., Immunity 2006	https://www.ncbi.nlm.nih.gov/pubmed/16901729
Th2	Hif1a	Okoye IS et al., Proc Natl Acad Sci	https://www.ncbi.nlm.nih.gov/pubmed/25024218
Th2	Hivep2	Nakayama T et al., Adv Exp Med Biol 2010	https://www.ncbi.nlm.nih.gov/pubmed/20795536
Th2	Il10	Fiorentino DF et al., J Exp Med 1989	https://www.ncbi.nlm.nih.gov/pubmed/2531194
Th2	Il13	O'Garra A, Immunity 1998	https://www.ncbi.nlm.nih.gov/pubmed/9529145
Th2	Il1r2	Okoye IS et al., Proc Natl Acad Sci	https://www.ncbi.nlm.nih.gov/pubmed/25024218
Th2	Il1rl1	Guo L et al., Nat Immunol 2015	https://www.ncbi.nlm.nih.gov/pubmed/26322482
Th2	Il2	Yokyama A et al., Immunol Lett 1989	https://www.ncbi.nlm.nih.gov/pubmed/2570041
Th2	Il24	Lin CC et al., Nat Commun 2014	https://www.ncbi.nlm.nih.gov/pubmed/24699451
Th2	Il2ra	Cote-Sierra J et al., Proc Natl Acad Sci 2004	https://www.ncbi.nlm.nih.gov/pubmed/15004274
Th2	Il2rb	Cote-Sierra J et al., Proc Natl Acad Sci 2004	https://www.ncbi.nlm.nih.gov/pubmed/15004274
Th2	Il4	O'Garra A, Immunity 1998	https://www.ncbi.nlm.nih.gov/pubmed/9529145
Th2	Il4ra	Liao W et al., Nat Immunol 2008	https://www.ncbi.nlm.nih.gov/pubmed/18820682
Th2	Il5	O'Garra A, Immunity 1998	https://www.ncbi.nlm.nih.gov/pubmed/9529145
Th2	Irf4	Bruchard M et al., Nat Immunol 2015	https://www.ncbi.nlm.nih.gov/pubmed/26098997
Th2	Itgb2	Salomon B et al., J Immunol 1998	https://www.ncbi.nlm.nih.gov/pubmed/9820482
Th2	Itgb3	Horiuchi S et al., J Immunol 2011	https://www.ncbi.nlm.nih.gov/pubmed/21536806
Th2	Itk	Miller AT et al., Immunity 2004	https://www.ncbi.nlm.nih.gov/pubmed/15345221
Th2	Jak3	Ashino S et al., J Allergy Clin Immunol 2014	https://www.ncbi.nlm.nih.gov/pubmed/24365136
Th2	Jdp2	Horiuchi S et al., J Immunol 2011	https://www.ncbi.nlm.nih.gov/pubmed/21536806
Th2	Junb	Li B et al., EMBO J 1999	https://www.ncbi.nlm.nih.gov/pubmed/9889198
Th2	Lfng	Gu W et al., Plos One 2012	https://www.ncbi.nlm.nih.gov/pubmed/23071776
Th2	Maf	Ho IC et al., J Exp Med 1998	https://www.ncbi.nlm.nih.gov/pubmed/9815263
Th2	Nbr1	Yang JQ et al., EMBO J 2010	https://www.ncbi.nlm.nih.gov/pubmed/20808283
Th2	Nfil3	Okoye IS et al., Proc Natl Acad Sci	https://www.ncbi.nlm.nih.gov/pubmed/25024218
Th2	Nfkb1	Das J et al., Nat Immunol 2001	https://www.ncbi.nlm.nih.gov/pubmed/11135577
Th2	Nlrp3	Bruchard M et al., Nat Immunol 2015	https://www.ncbi.nlm.nih.gov/pubmed/26098997
Th2	Notch1	Amen D et al., Cell 2004	https://www.ncbi.nlm.nih.gov/pubmed/15137944
Th2	Pcgf2	Kimura M et al., Immunity 2001	https://www.ncbi.nlm.nih.gov/pubmed/11520462
Th2	Pparg	Saubermann LJ et al., Inflamm Bowel Dis 2002	https://www.ncbi.nlm.nih.gov/pubmed/12479648
Th2	Prdm1	Martins G et al., Annu Rev Immunol 2008	https://www.ncbi.nlm.nih.gov/pubmed/18370921
Th2	Prkcq	Cannons JL et al., Immunity 2004	https://www.ncbi.nlm.nih.gov/pubmed/15539155
Th2	Pros1	Chan PY et al., Science 2016	https://www.ncbi.nlm.nih.gov/pubmed/27034374
Th2	Ptgir	Zhou W et al., J Leukoc Biol 2007	https://www.ncbi.nlm.nih.gov/pubmed/17135575
Th2	Rbpj	Amen D et al., Cell 2004	https://www.ncbi.nlm.nih.gov/pubmed/15137944
Th2	Rgs1	Okoye IS et al., Proc Natl Acad Sci	https://www.ncbi.nlm.nih.gov/pubmed/25024218
Th2	Rictor	Yang K et al., Immunity 2013	https://www.ncbi.nlm.nih.gov/pubmed/24315998
Th2	Rnf128	Horiuchi S et al., J Immunol 2011	https://www.ncbi.nlm.nih.gov/pubmed/21536806
Th2	Rsad2	Qiu LQ et al., Blood 2009	https://www.ncbi.nlm.nih.gov/pubmed/19047684
Th2	S100a1	Horiuchi S et al., J Immunol 2011	https://www.ncbi.nlm.nih.gov/pubmed/21536806
Th2	Socs2	Okoye IS et al., Proc Natl Acad Sci	https://www.ncbi.nlm.nih.gov/pubmed/25024218
Th2	Socs3	Egwuagu CE et al., J Immunol 2002	https://www.ncbi.nlm.nih.gov/pubmed/11907070
Th2	Stat5a	Zhu J et al., Immunity 2003	https://www.ncbi.nlm.nih.gov/pubmed/14614860
Th2	Stat5b	Zhu J et al., Immunity 2003	https://www.ncbi.nlm.nih.gov/pubmed/14614860
Th2	Stat6	Kaplan MH et al., Immunity 1996	https://www.ncbi.nlm.nih.gov/pubmed/8624821
Th2	Stx11	Okoye IS et al., Proc Natl Acad Sci	https://www.ncbi.nlm.nih.gov/pubmed/25024218
Th2	Tanc2	Horiuchi S et al., J Immunol 2011	https://www.ncbi.nlm.nih.gov/pubmed/21536806
Th2	Tmtc2	Sasaki T et al., Plos One 2013	https://www.ncbi.nlm.nih.gov/pubmed/23824597
Th2	Tnfrsf4	Ito T et al., J Exp Med 2005	https://www.ncbi.nlm.nih.gov/pubmed/16275760
Th2	Tnfrsf8	Romagnani S et al., J Leukoc Biol 1995	https://www.ncbi.nlm.nih.gov/pubmed/7759952
Th2	Tnfrsf9	Pollok KE et al., J Immunol 1993	https://www.ncbi.nlm.nih.gov/pubmed/7678621
Th2	Tube1	Horiuchi S et al., J Immunol 2011	https://www.ncbi.nlm.nih.gov/pubmed/21536806
Th2	Vav1	Capitani N et al., J allergy Clin Immunol 2010	https://www.ncbi.nlm.nih.gov/pubmed/20638113
Th17	Ahr	Quintana FJ et al., Nature 2008	https://www.ncbi.nlm.nih.gov/pubmed/18362915
Th17	Anxa1	Gavins FN et al., Front Immunol 2012	https://www.ncbi.nlm.nih.gov/pubmed/23230437
Th17	Arrb1	Li J et al., Prco Natl Acad Sci 2013	https://www.ncbi.nlm.nih.gov/pubmed/23589893
Th17	Batf	Schraml BU et al., Nature 2009	https://www.ncbi.nlm.nih.gov/pubmed/19578362
Th17	Cd274	Hirahara K et al., Immunity 2012	https://www.ncbi.nlm.nih.gov/pubmed/22726954
Th17	Il17a	Liang SC et al., J Exp Med 2006	https://www.ncbi.nlm.nih.gov/pubmed/16982811
Th17	Il17f	Liang SC et al., J Exp Med 2006	https://www.ncbi.nlm.nih.gov/pubmed/16982811
Th17	Il17ra	Walinne CC et al., Genes Immun 2011	https://www.ncbi.nlm.nih.gov/pubmed/20861865
Th17	Il1r1	Sutton C et al., J Exp Med 2006	https://www.ncbi.nlm.nih.gov/pubmed/16818675
Th17	Il21	Nurieva R et al., Nature 2007	https://www.ncbi.nlm.nih.gov/pubmed/17581589

Th17	Il21r	Nurieva R et al., Nature 2007	https://www.ncbi.nlm.nih.gov/pubmed/17581589
Th17	Il23r	Zhou L et al., Nat Immunol 2007	https://www.ncbi.nlm.nih.gov/pubmed/17581537
Th17	Il6ra	Ciofani M et al., Cell 2012	https://www.ncbi.nlm.nih.gov/pubmed/23021777
Th17	Irf4	Brüstle A et al., Nat Immunol 2007	https://www.ncbi.nlm.nih.gov/pubmed/17676043
Th17	Itgb2	Rothhammer V et al., J Exp Med 2011	https://www.ncbi.nlm.nih.gov/pubmed/22025301
Th17	Jak2	Conti L et al., J Immunol 2012	https://www.ncbi.nlm.nih.gov/pubmed/22219326
Th17	Junb	Yamazaki S et al., Scientific Reports 2017	https://www.ncbi.nlm.nih.gov/pubmed/29234109
Th17	Klf4	Lebson L et al., J Immunol 2010	https://www.ncbi.nlm.nih.gov/pubmed/21076063
Th17	Mapk9	Ichiyama K et al., Immunity 2011	https://www.ncbi.nlm.nih.gov/pubmed/21600798
Th17	Nbr1	Yang QC et al., J Immunol 2013	http://www.jimmunol.org/content/190/1_Supplement/65.1
Th17	Notch1	Keerthivasan S et al., J Immunol 2011	https://www.ncbi.nlm.nih.gov/pubmed/21685328
Th17	Notch2	Keerthivasan S et al., J Immunol 2011	https://www.ncbi.nlm.nih.gov/pubmed/21685328
Th17	Nr4a2	Raveney BJ et al., Plos One 2013	https://www.ncbi.nlm.nih.gov/pubmed/23437182
Th17	P2rx7	Fernandez D et al., Plos One 2016	https://www.ncbi.nlm.nih.gov/pubmed/27322617
Th17	Pcgf2	Hod-Dvorai R et al., Eur J Immunol 2011	https://www.ncbi.nlm.nih.gov/pubmed/21674483
Th17	Prdm1	Jain R et al., Immunity 2016	https://www.ncbi.nlm.nih.gov/pubmed/26750311
Th17	Prkcq	Kwong MJ et al., J Immunol 2012	https://www.ncbi.nlm.nih.gov/pubmed/22586032
Th17	Rel	Cheng G et al., J Immunol 2011	https://www.ncbi.nlm.nih.gov/pubmed/21940679
Th17	Rela	Ruan Q et al., J Exp Med 2011	https://www.ncbi.nlm.nih.gov/pubmed/22006976
Th17	Rora	Yang X et al., Immunity 2008	https://www.ncbi.nlm.nih.gov/pubmed/18164222
Th17	Rorc	Ivanov II et al., Cell 2006	https://www.ncbi.nlm.nih.gov/pubmed/16990136
Th17	Runx1	Zhang F et a., Nat Immunol 2008	https://www.ncbi.nlm.nih.gov/pubmed/18849990
Th17	Slc3a2	Cantor J et al., J Immunol 2011	https://www.ncbi.nlm.nih.gov/pubmed/21670318
Th17	Smad2	Veldhoen M et al., Nat Immunol 2006	https://www.ncbi.nlm.nih.gov/pubmed/16998492
Th17	Smad3	Veldhoen M et al., Nat Immunol 2006	https://www.ncbi.nlm.nih.gov/pubmed/16998492
Th17	Socs3	Taleb S et al., J Exp Med 2009	https://www.ncbi.nlm.nih.gov/pubmed/19737863
Th17	Stat3	Harry TJ et al., J Immunol 2007	https://www.ncbi.nlm.nih.gov/pubmed/17878325
Th17	Tgfbr1	Veldhoen M et al., Nat Immunol 2006	https://www.ncbi.nlm.nih.gov/pubmed/16998492
Th17	Tgfbr2	Veldhoen M et al., Nat Immunol 2006	https://www.ncbi.nlm.nih.gov/pubmed/16998492
Th17	Tyk2	Ishizaki M et al., J Immunol 2011	https://www.ncbi.nlm.nih.gov/pubmed/21606247
Th17	Usp18	Liu X et al., J Exp Med 2013	https://www.ncbi.nlm.nih.gov/pubmed/23825189
Th17	Vav1	Kassem S et al., Plos Genetics 2016	https://www.ncbi.nlm.nih.gov/pubmed/27438086

Supplementary Figure 1

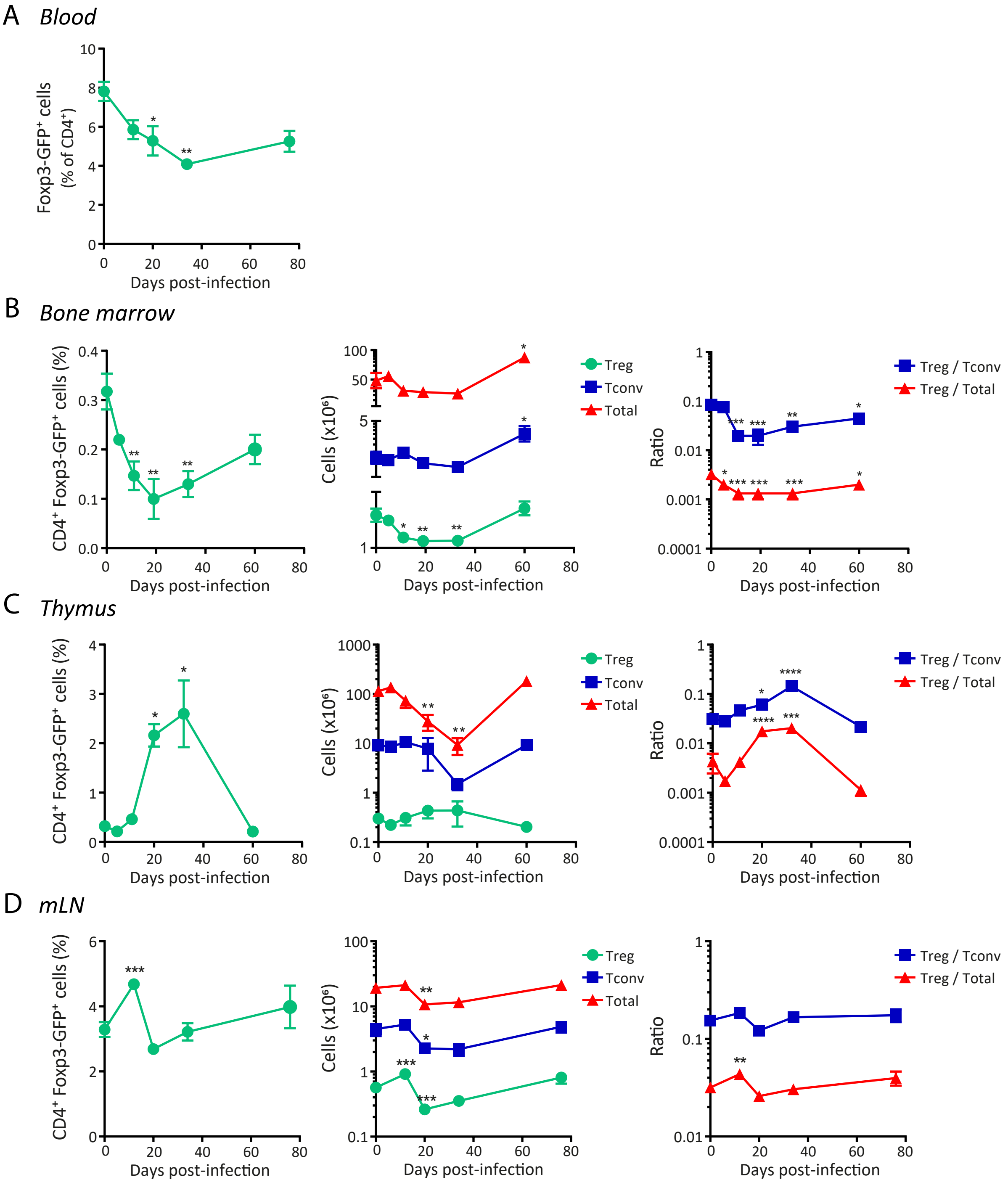

Supplementary Figure 1: Reduced frequency of Treg cells in periphery but not in organs of Treg cell development during acute *T. cruzi* infection. A-D) Graphs showing CD4+ Foxp3-GFP+ Treg cell frequencies (left panel), absolute numbers of Treg cells, CD4+ Foxp3-GFP- (Tconv) cells and total leukocytes (middle panel), and the ratios of Treg cells to Tconv and to total cells (right panel) in blood (**A**), bone marrow (**B**), thymus (**C**) and mesenteric lymph nodes (mLN) (**D**) of *T. cruzi* infected Foxp3-GFP mice at different dpi. Data are presented as mean ± SEM, n=2-11 depending on the dpi and the organ. Data are representative of 3 independent experiments for thymus, 2 independent experiments for blood and mLN and 1 experiment for bone marrow. P values were calculated by One way ANOVA with Dunnett's multiple comparisons test or Kruskal-Wallis with Dunn's correction (Treg cells frequencies in blood and thymus only). * $P \leq 0.05$, ** $P \leq 0.01$, *** $P \leq 0.001$ and **** $P \leq 0.0001$.

Supplementary figure 2

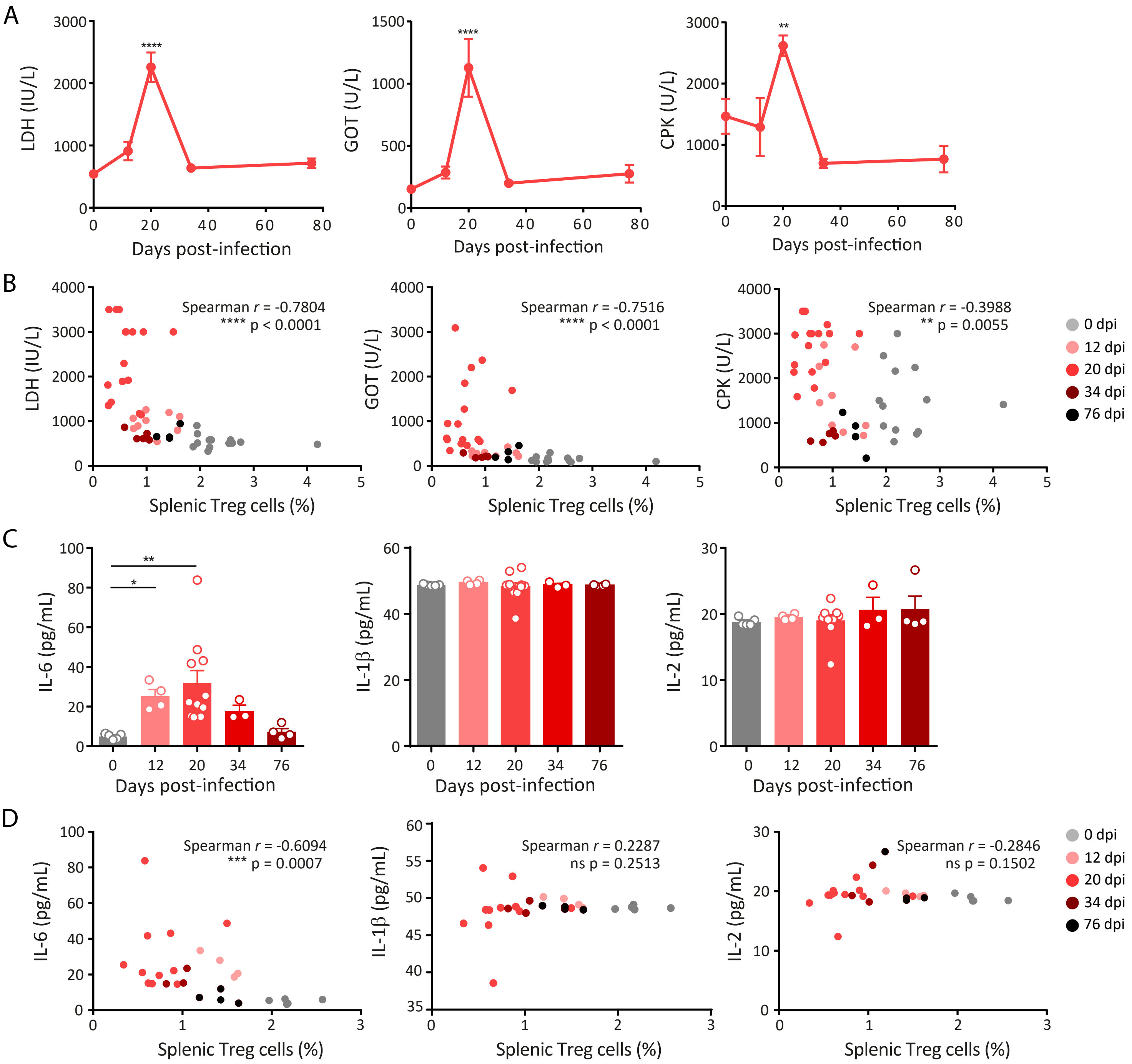

Supplementary Figure 2: Treg cells frequency inversely correlates with biochemical markers of tissue damage and effector cytokines in blood. A) Activity of lactate dehydrogenase (LDH) (left panel), glutamate-oxalacetic transaminase (GOT) (middle panel) and creatine phosphokinase (CPK) (right panel) in plasma of *T. cruzi* infected Foxp3-GFP mice at different dpi. **B)** Scatter plots showing the relation between splenic Treg cell frequencies and each of the parameters shown in A). **C)** Effector cytokines concentration in plasma of *T. cruzi* infected Foxp3-EGFP mice at different dpi. **D)** Scatter plots showing the relation between splenic Treg cell frequencies and plasma level of effector cytokines. In A) data are presented as mean ± SEM of n=3-11 animals. In B), C) and D) each circle or dot represents one animal. In C) bars show the mean + SEM of each cytokine level. Spearman *r* correlation coefficient and significance of the correlation are indicated inside the corresponding graphs. Data were pooled from 2 independent experiments for 20 dpi and correspond to one experiment for other dpi. P values were calculated by One way ANOVA with Dunnett's multiple comparisons test or Kruskal-Wallis with Dunn's correction (IL-6 levels only). * P ≤ 0.05, ** P ≤ 0.01, *** P ≤ 0.001, **** P ≤ 0.0001 and ns = not significant.

Supplementary figure 3

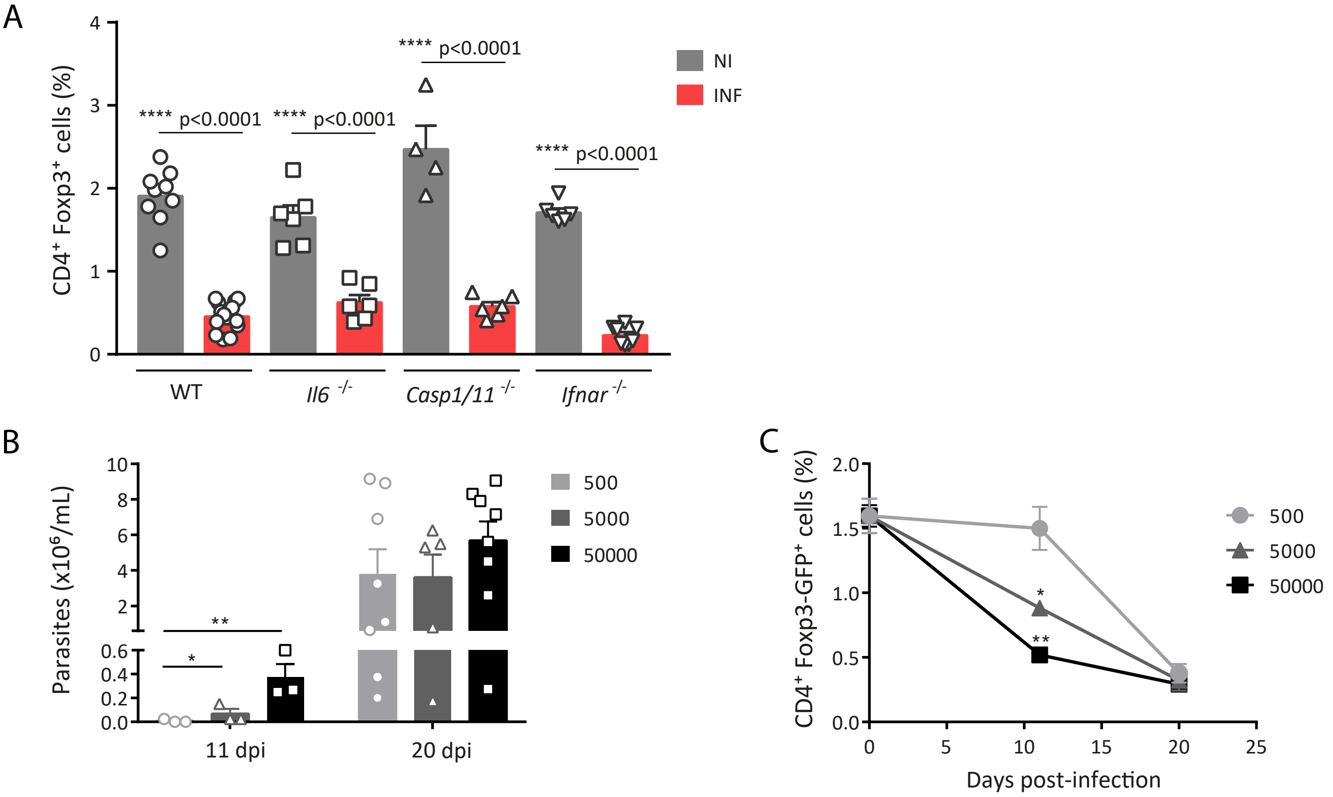

Supplementary Figure 3: Reduced Treg cell frequency in periphery during *T. cruzi* infection is not induced by inflammatory cytokines but correlates with parasite levels. A) Percentages of Treg cells (CD4+ Foxp3+) in the spleens of non-infected (NI) or infected (INF, 18 dpi) WT animals and mice deficient in IL-6, Caspase1/11 or IFNAR. Data are pooled from at least 2 independent experiments. **B-C)** Parasitemia (**B**) and frequencies of splenic Treg cells (**C**) determined at 11 and 20 dpi in Foxp3-EGFP mice infected with different doses of *T. cruzi*: 500, 5000 (usual) and 50000 trypomastigotes (tp). P values were calculated by One Way ANOVA with Tukey's correction. In C) stars stand for comparison between the dose of 500 tp and the doses of 5000 and 50000 tp. * P ≤ 0.05, ** P ≤ 0.01 and **** P ≤ 0.0001.

Supplementary figure 4

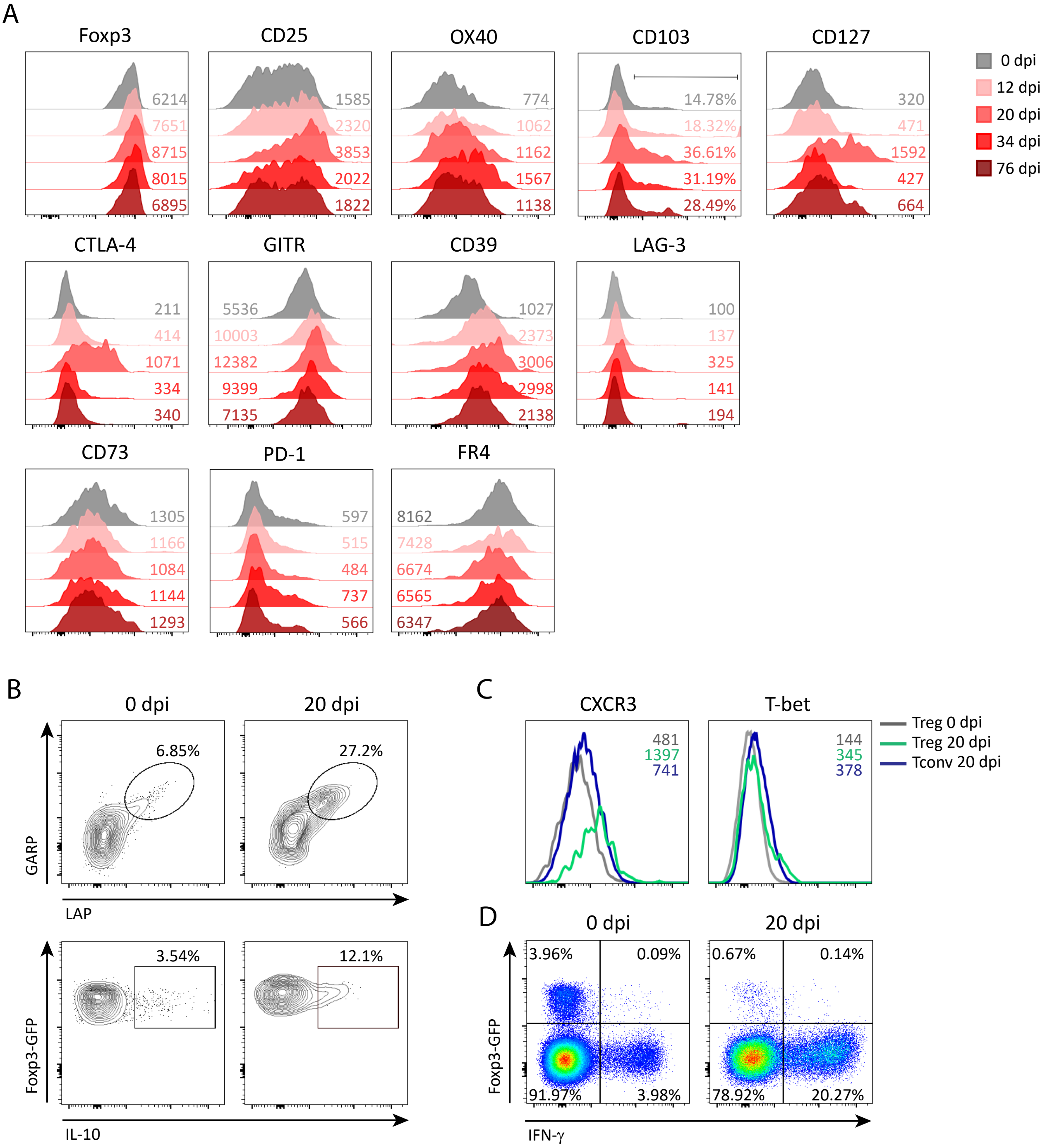

Supplementary figure 4: Expression of Treg cell markers of activation, suppressive function and Th1 specialization during *T. cruzi* infection. A) Histograms illustrating the expression of different markers of activation and suppressive function in Treg cells from the spleen of Foxp3-EGFP mice at different dpi. Numbers inside the plots indicate geometric mean of fluorescence intensity or percentage of positive cells. **B)** Dot plots showing LAP+ GARP+ double positive or IL-10 producing Treg cells (CD4+ Foxp3-GFP+) from the spleen of non-infected (0 dpi) Foxp3-EGFP mice and 20 days post- *T. cruzi* infection (20 dpi). **C)** Histograms showing CXCR3 and T-bet expression in Treg and Tconv cells from the spleen of non-infected (0 dpi) and/or 20-day-infected Foxp3-EGFP mice. Numbers inside the plots indicate geometric mean of fluorescence intensity. **D)** Dot plots showing Foxp3-GFP expression and IFN-γ staining in CD4+ T cells from the spleen of non-infected (0 dpi) and 20-day-infected Foxp3-EGFP mice. Graphs are representative of 9-11 mice. Similar results were obtained in at least 2 independent experiments.

Supplementary figure 5

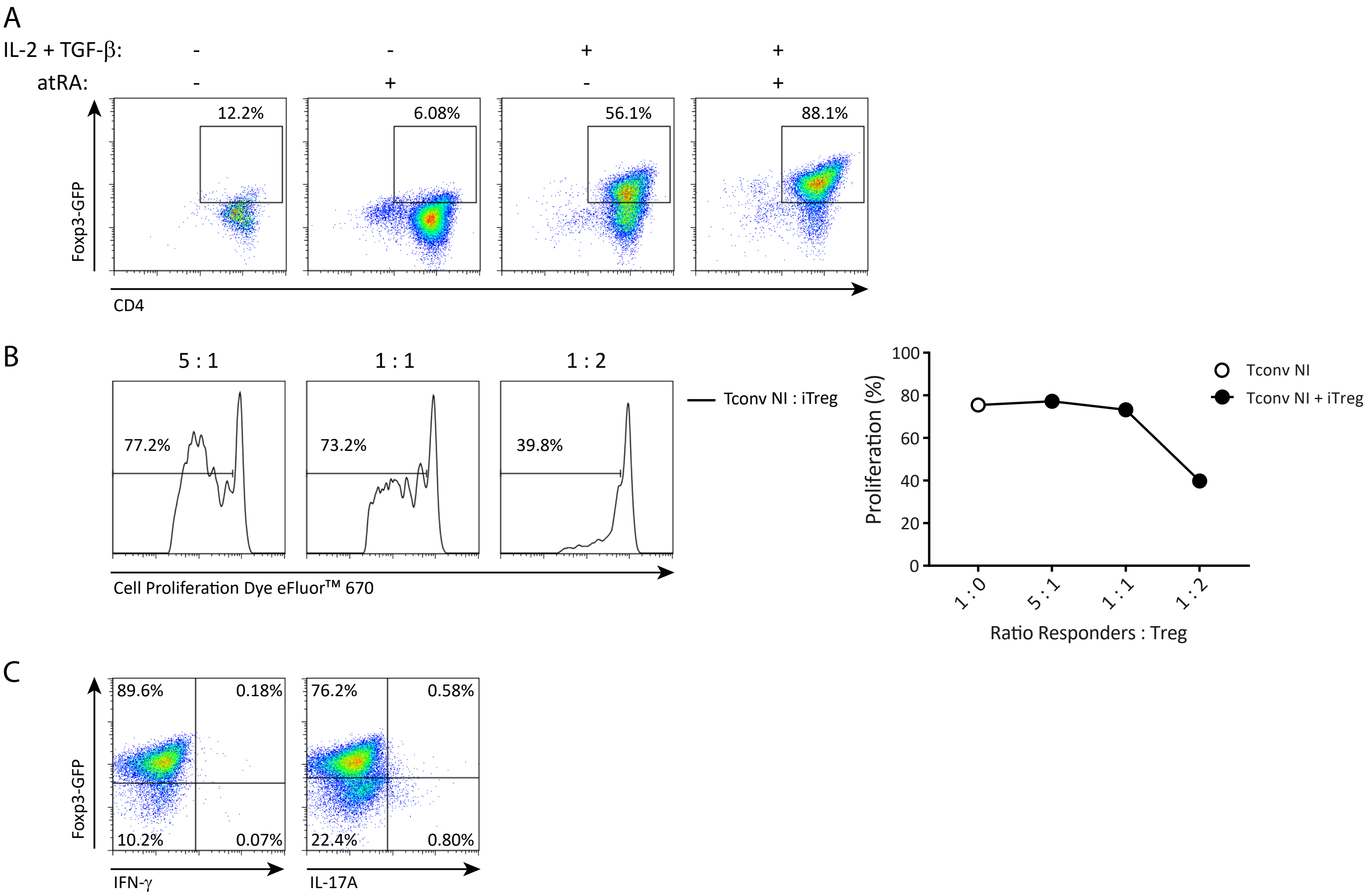

Supplementary Figure 5: Functional characterization of *in vitro* differentiated Treg cells and adoptive transfer complementary data. A-C) *In vitro* differentiation of Treg cells. Naïve CD4+ T cells (CD4+ Foxp3-GFP- CD25- CD62L+ CD44-) purified from the spleens of non-infected Foxp3-EGFP CD45.2 mice were cultured for four days in the presence or absence of IL-2, TGF-β, *all-trans* Retinoic Acid (atRA), and plate-bound anti-CD3 and anti-CD28. Dot plots illustrating percentage of Treg cells generated after four days under the different differentiation conditions (A). *In vitro* differentiated Treg (iTreg) cells were purified by cell sorting as Foxp3-GFP+ and cultured in different ratios with responders Tconv cells purified from non-infected Foxp3-EGFP mice and labeled with Cell Proliferation Dye eFluor™ 670. Four days later, proliferation of responder cells was assessed by dilution of Dye eFluor™ 670 (B). Production of effector cytokines was evaluated by flow cytometry in iTreg cells (C).
